# Effect of maternal prenatal and postpartum vitamin D supplementation on offspring bone mass and muscle strength in early childhood: follow-up of a randomized controlled trial

**DOI:** 10.1093/ajcn/nqab396

**Published:** 2021-11-27

**Authors:** Karen M O'Callaghan, Shaila S Shanta, Farzana Fariha, Jennifer Harrington, Abdullah Al Mahmud, Abby L Emdin, Alison D Gernand, Tahmeed Ahmed, Steven A Abrams, Daniel R Moore, Daniel E Roth

**Affiliations:** Centre for Global Child Health and SickKids Research Institute, Hospital for Sick Children, Toronto, Ontario, Canada; Nutrition and Clinical Services Division, International Centre for Diarrhoeal Disease Research, Bangladesh, Dhaka, Bangladesh; Nutrition and Clinical Services Division, International Centre for Diarrhoeal Disease Research, Bangladesh, Dhaka, Bangladesh; Department of Pediatrics, Hospital for Sick Children and University of Toronto, Toronto, Ontario, Canada; Nutrition and Clinical Services Division, International Centre for Diarrhoeal Disease Research, Bangladesh, Dhaka, Bangladesh; Centre for Global Child Health and SickKids Research Institute, Hospital for Sick Children, Toronto, Ontario, Canada; Department of Nutritional Sciences, The Pennsylvania State University, State College, PA, USA; Nutrition and Clinical Services Division, International Centre for Diarrhoeal Disease Research, Bangladesh, Dhaka, Bangladesh; Department of Pediatrics, Dell Medical School at the University of Texas at Austin, Austin, TX, USA; Faculty of Kinesiology and Physical Education, University of Toronto, Toronto, Ontario, Canada; Centre for Global Child Health and SickKids Research Institute, Hospital for Sick Children, Toronto, Ontario, Canada; Department of Pediatrics, Hospital for Sick Children and University of Toronto, Toronto, Ontario, Canada

**Keywords:** bone mineral content, areal bone mineral density, grip strength, randomized controlled trial, vitamin D

## Abstract

**Background:**

Maternal vitamin D status during pregnancy and lactation is a modifiable factor that may influence offspring musculoskeletal outcomes. However, few randomized trials have tested the effects of prenatal or postpartum vitamin D supplementation on offspring bone and muscle development.

**Objectives:**

The aim was to examine hypothesized effects of improvements in early-life vitamin D status on childhood musculoskeletal health in Dhaka, Bangladesh.

**Methods:**

In a previously completed, double-blind, dose-ranging trial, healthy pregnant women (*n* = 1300) were recruited at 17–24 weeks’ gestation and randomly assigned to a prenatal/postpartum regimen of 0/0, 4200/0, 16,800/0, 28,000/0, or 28,000/28,000 IU cholecalciferol (vitamin D_3_)/wk until 26 wk postpartum. In this new report, we describe additional follow-up at 4 y of age (*n* = 642) for longer-term outcomes. Bone mineral content (BMC) and areal bone mineral density (aBMD) were measured by DXA. Grip strength was tested using a hand-held dynamometer. The primary comparison was children of women assigned to 28,000 IU/wk prenatally compared with placebo. Differences are expressed as means and 95% CIs.

**Results:**

Total-body-less-head (TBLH) BMC, TBLH aBMD, and grip strength were similar in the combined high-dose prenatal (28,000/0 and 28,000/28,000 IU/wk) compared with placebo groups (mean difference [95% CI] = 0.61 g [–10.90, 12.13], 0.0004 g/cm^2^ [–0.0089, 0.0097], and 0.02 kg [–0.26, 0.31], respectively). In dose-ranging analyses, TBLH BMC and aBMD, whole-body BMC and aBMD, and grip strength in each of the prenatal vitamin D groups were not significantly different from placebo (*P* > 0.05 for all comparisons). Only head aBMD was greater in children of women assigned to the 28,000/28,000-IU regimen compared with placebo (mean difference [95% CI] = 0.024 g/cm^2^ [0.0009, 0.047], *P* = 0.042); the effect was attenuated upon adjustment for child height, weight, and sex (*P* = 0.11).

**Conclusions:**

Maternal prenatal, with or without postpartum, vitamin D supplementation does not improve child BMC, aBMD, or grip strength at 4 y of age. The MDIG trial and present follow-up study were registered prospectively at www.clinicaltrials.gov as NCT01924013 and NCT03537443, respectively.

## Introduction

Low bone mass, reflected by a low bone mineral content (BMC) and low bone mineral density (BMD), contributes to fracture risk in childhood (1) and osteoporotic fractures in later life (2). Bone mass tracks longitudinally from childhood to late adolescence, with slower rates of early accrual contributing to an overall lower peak bone mass (3, 4). Targeted interventions that maximize early-life bone mineral accrual may therefore reduce risks of fracture in childhood as well as prevent osteopenia and osteoporosis later in life (3).

The importance of vitamin D for the promotion and maintenance of bone health throughout the life cycle has been well established (5, 6). Isolation of the vitamin D receptor in skeletal muscle suggests an additional role for vitamin D in muscle development and functioning (7, 8), consistent with the association of muscular weakness (9, 10) and motor delay (11, 12) in children and adolescents with vitamin D deficiency. As a clinical feature of rickets and osteomalacia, proximal myopathy contributes to a reduction in functional strength that is reversible upon treatment with vitamin D (10, 13, 14). Maternal vitamin D status during pregnancy may be a modifiable contributor to offspring musculoskeletal health (15), yet observational studies have shown inconsistent evidence of an association with neonatal (16), childhood (17–19), or peak bone mass (20). Only a few trials have directly tested the effects of prenatal vitamin D supplementation on offspring bone mass accrual or body composition (21–24) or muscle strength in childhood (23). Although observational data from the United Kingdom have shown a positive association between maternal prenatal vitamin D status and offspring grip strength (25), evidence for effects of vitamin D on offspring bone and muscle outcomes is particularly limited for populations with habitually low circulating 25-hydroxyvitamin D [25(OH)D] concentrations (biomarker of vitamin D status), who may be considered most likely to benefit from routine vitamin D supplementation.

In a population with a high prevalence of maternal and neonatal vitamin D deficiency [serum 25(OH)D <30 nmol/L], we previously found that prenatal and postpartum vitamin D supplementation substantially increased maternal and infant 25(OH)D but did not affect infant linear growth (26). We further hypothesized that improvements in fetal and infant vitamin D status in this cohort would increase bone mineralization, rather than bone length, with complementary effects on the developing muscle. In the present follow-up study at 4 y of age, we aimed to test the effect of prenatal vitamin D supplementation, with and without postpartum supplementation, on DXA-derived measures of offspring BMC and areal BMD (aBMD), as well as hand-grip strength and other DXA-derived measures of body composition.

## Methods

### Study design

The BONe and mUScle health in Kids (BONUSKids) study was an observational follow-up of a double-blind, dose-ranging trial of maternal cholecalciferol (vitamin D_3_) supplementation [Maternal Vitamin D for Infant Growth (MDIG)] trial in Dhaka, Bangladesh, for which the methods and primary outcomes were previously reported (26, 27). Briefly, generally healthy women (*n* = 1300) with uncomplicated singleton pregnancies were enrolled at 17–24 weeks’ gestation and randomly assigned to 1 of 5 trial arms comprising a prenatal/postpartum regimen of 0/0, 4200/0, 16,800/0, 28,000/0, or 28,000/28,000 IU vitamin D_3_/wk until 6 mo postpartum. In addition to the intervention dose, all participants were provided with standard iron-folic acid and calcium supplementation (500 mg/d as calcium carbonate) from enrollment to 6 mo postpartum. Births occurred from June 2014 through to February 2016, with postnatal follow-up assessments at 2 y of age completed in March 2018.

MDIG trial participants who expressed interest in participating in future sub-studies were contacted to request their child's participation in BONUSKids. Eligibility was determined upon availability for participation at 45 to 51 mo of age and maternal adherence to the assigned intervention dose during the prenatal period (≥80% of assigned tablets). Children were ineligible if unable to ambulate without assistance, supported by an orthopedic cast, or diagnosed with a developmental disorder that would limit feasible DXA scanning. BONUSKids study activities began in October 2018 and occurred on a rolling basis until February 2020.

### Ethics

Ethical approval was obtained from the Research Ethics Committees of the Hospital for Sick Children in Toronto [Research Ethics Board (REB) no. 1000060961] and the International Centre for Diarrhoeal Disease Research, Bangladesh (icddr,b; PR-18041). Written informed consent was provided by all women prior to commencing the MDIG trial, and additional consent was provided by caregivers for their child's participation in BONUSKids at 4 y of age. The MDIG trial (ID: NCT01924013) and present follow-up study (ID: NCT03537443) were registered prospectively at www.clinicaltrials.gov.

### Sample size and power calculations

The target sample size was based on the primary objective of detecting a meaningful difference in BMC at 4 y of age between the placebo and combined high-dose (28,000 IU/wk prenatally with or without 28,000 IU/wk postpartum) supplementation groups. We estimated that 120 children from each intervention group (120 placebo, 240 high-dose supplementation) would yield 80% power to detect a 22-g [standardized mean difference (SMD) = 0.31] difference in mean BMC and 90% power to detect a 25-g (SMD = 0.36) difference in BMC, given a 2-sided 5% type I error rate and assuming a CV of 13%, as per Hazell et al. (28). Allowing for 15% attrition, the target sample size was raised to 140 children from each of the 5 groups in the original trial, expecting an even distribution across trial arms given the randomized design. Additional information on power calculations for secondary outcomes is provided as **Supplemental Methods**.

### Anthropometry

Height at 4 y was measured to the last completed 1 mm using a stadiometer (Leicester Height Measure; Chasmors). Weight was measured to the nearest 50 g on a digital scale (Seca 874; Seca). Duplicate measures of both height and weight were taken, with a repeated set of measures for any discrepancy of ≥1 cm in height and ≥50 g in weight. Mean values of acceptable paired measures were used for analysis. Anthropometric *z* scores (height-, weight-, and BMI-for-age) were calculated according to the WHO child growth standards (29).

### DXA

Full-body DXA scans were performed by trained technologists at a collaborating health facility (Popular Diagnostic Centre, Dhanmondi, Dhaka, Bangladesh) on a GE Lunar Prodigy narrow-angle fan-beam DXA scanner (GE Healthcare) using the enhanced analysis mode and enCORE software version 16.0. Quality assurance was performed by daily calibration using a standard block phantom prior to participant scanning. DXA images of the total-body-less-head (TBLH; subcranial skeleton from base of neck to feet), the head, and whole body (WB; head to feet) were assessed independently by 2 reviewers for motion artifact and scan quality and categorized into 1 of 3 mutually exclusive groups: *1*) no (or negligible) motion artifact, *2*) minor motion artifact (including DXA-imputed estimates of unintentionally omitted contralateral sites), and *3*) major motion artifact (Supplemental Methods). Discrepancies were resolved by a third reviewer to determine the DXA scans for inclusion in primary analysis. TBLH BMC and aBMD *z* scores were calculated using the Lambda-Mu-Sigma (LMS)–modeled formulas proposed by Crabtree et al. (30).

### Grip strength

Grip strength was measured with a hand-held digital dynamometer (Jamar; Patterson Medical), using a standardized approach (31). All measurements were conducted in a seated position with the active arm resting at a 90° angle. Three measurements were taken for each hand, recorded to the nearest 100 g, giving a total target of 6 measurements. A 30-s break was taken between each attempt to avoid fatigue, calculated by a digital stopwatch. Both the mean and maximum values of all attempted measurements were calculated; the maximum value was decided a priori for use in main analyses (31).

### Laboratory analyses

Maternal serum 25(OH)D was measured at enrollment and delivery by high-performance LC-MS/MS at the Analytical Facility for Bioactive Molecules (Hospital for Sick Children, Toronto, Canada), as described previously (26). The lower limit of quantification (LLoQ) for both 25-hydroxyvitamin D_3_ [25(OH)D_3_] and 25-hydroxyvitamin D_2_ [25(OH)D_2_] was 1.25 nmol/L; 25(OH)D_2_ concentrations were undetectable or negligible in this cohort and hence not reported. Analysis of 25(OH)D at 4 y of age was performed using a similar approach (26), including chromatographic separation and quantification of 25(OH)D_3_, 3-epi-25-hydroxyvitamin D_3_ [3-epi-25(OH)D_3_], and 25(OH)D_2_, and use of National Institute of Standards and Technology (NIST) quality-control materials (SRM 972a) and Vitamin D External Quality Assessment Scheme (DEQAS) standards. The LLoQ for 25(OH)D_3_ at 4 y was 0.05 nmol/L. The LLoQ for 25(OH)D_2_ was 0.125 nmol/L; however, as 25(OH)D_2_ concentrations were undetectable in all participants, only concentrations of 25(OH)D_3_ are reported in the present analysis, excluding 3-epi-25(OH)D_3._ Average bias and interassay CV for 25(OH)D_3_ at 4 y of age was −9.3% and 9.0%, respectively.

### Outcomes

Consistent with recommendations from the International Society for Clinical Densitometry (32), bone mass outcomes were primarily quantified as TBLH BMC and aBMD as TBLH measures are considered more responsive to environmental exposures (33) and have higher reproducibility compared with WB measures (34). Although BMC was appointed as the primary outcome based on previous recommendations (35, 36), BMC and aBMD are closely related and complementary measures of bone mass, and therefore both outcomes are reported in all analyses. Secondary outcomes included WB BMC and aBMD measurements, BMC and aBMD measurements of the head alone, and analyses with covariate adjustment for height, weight, and sex. Nonskeletal secondary outcomes included hand-grip strength and DXA-derived TBLH and WB absolute fat and lean mass (kilograms), and fat tissue mass percentage (i.e., proportion of fat relative to nonosseous lean mass). Fat-free mass (FFM) was calculated as the sum of (TBLH or WB) lean mass and BMC. Fat mass index (FMI) was calculated as (TBLH or WB) fat mass (kilograms) divided by height in meters squared.

Given the randomized study design, and because previous analyses showed no effect of the vitamin D intervention on linear or ponderal growth up to 12 mo of age (26), we expected to find comparable average heights and weights across intervention groups at 4 y. Therefore, we decided a priori to use unadjusted models for the primary analytical approach based on the likelihood that concurrent height/weight is more likely to mediate rather than confound the effects of the vitamin D intervention on musculoskeletal outcomes.

### Statistical analysis

For DXA-derived measures (BMC, aBMD, fat and lean mass, and fat mass percentage), primary analyses were completed using values obtained from DXA scans showing correct alignment and either no or minor motion only, according to the criteria outlined in the Supplemental Methods. To examine whether effect estimates were influenced by slight motion artifact, sensitivity analyses were explored using data from DXA reports without any movement or incorrect alignment (Supplemental Methods).

### Data distributions and descriptive statistics

Data distributions were visually inspected using histograms and kernel density plots. Bivariate relations between each outcome and prenatal supplemental vitamin D intake (as a continuous variable) were assessed using scatterplots with locally weighted regression (LOWESS). Summary statistics are reported as means ± SDs or 95% CIs, median (minimum–maximum or IQR), or frequencies with percentages. To examine participant characteristics across intervention groups, we conducted either ANOVA with Tukey's post hoc or chi-square (χ^2^) tests for continuous or categorical variables, respectively. Where data departed from normality and nonparametric testing was appropriate, a Kruskal-Wallis test was used. Comparison of MDIG trial participants who enrolled in the BONUSKids follow-up study with those who did not participate was assessed using independent-samples *t* tests, Wilcoxon rank-sum tests, and χ^2^ tests, as appropriate. *P* < 0.05 was considered statistically significant.

### Placebo compared with high-dose prenatal vitamin D supplementation

In primary analyses, the 2 high-dose prenatal vitamin D groups were combined for a comparison of children of mothers who received 28,000 IU vitamin D/wk prenatally (with or without 28,000 IU/wk postpartum) to children whose mothers received placebo prenatally. Outcome measures were normally distributed and did not require transformation prior to analysis. Effects of the vitamin D interventions in primary analyses were examined by mean differences using independent 2-sided samples *t* tests.

### Dose-ranging effect of prenatal and postpartum vitamin D supplementation

To examine the dose-ranging association between vitamin D supplemental intake and offspring musculoskeletal health, we used data from all 5 allocated intervention groups, including disaggregation of the 2 high-dose prenatal vitamin D groups, to explore potential effects of prenatal-only compared with prenatal plus postpartum supplementation. Linear regression models were fitted using the assigned vitamin D dose as the (categorical) exposure variable and either BMC, aBMD, or grip strength as the (continuous) outcome variable. Estimates of the 95% CIs were obtained using a bootstrap procedure with 1000 replications. Similar analyses were conducted using fat mass (kilograms and %) and lean mass as the outcome variable to test effects of each intervention group on offspring body composition. In sensitivity dose–response analyses, we regressed each outcome on weekly supplemental vitamin D intake as a continuous exposure variable, using individual-level estimates of the prenatal dose received based on manufacturer analysis of the vitamin D tablet composition and accounting for maternal adherence to the intervention.

### Subgroup and auxiliary analyses

For each outcome [BMC, aBMD, grip strength, fat mass (kg and %), and lean mass], planned subgroup analyses included unadjusted regression models fitted to data stratified by child sex and maternal vitamin D status at randomization [25(OH)D ≥30 nmol/L vs. <30 nmol/L], which were further explored by considering the statistical interaction between sex and intervention group, and between maternal baseline 25(OH)D and intervention group for each outcome. Acknowledging a lower bone mineral accrual in utero (37) and risk of later metabolic bone disease among infants born premature (38), a post hoc decision was made to examine the effect of the intervention (prenatal, with or without postpartum, vitamin D supplementation) on each outcome using an analysis restricted to term-born infants (≥37 weeks’ gestation).

In further analyses, we included body-size measures (e.g., height and/or weight) as covariates to test their roles in mediating the association between the vitamin D intervention and child BMC, aBMD, grip strength, and body composition at 4 y of age. Additional post hoc sensitivity analyses were conducted to examine precision of the effect estimates for primary bone outcomes (TBLH BMC and aBMD) and maximum grip strength upon adjustment for the following selected early-life determinants of musculoskeletal health: maternal age and height, household asset index (as a proxy for socioeconomic status), and the duration of exclusive breastfeeding. Assuming data were missing at random, post hoc analyses using multiple imputation by chained equations (MICE) were performed for TBLH BMC, TBLH aBMD, and maximum grip strength including children for whom outcome data were missing in the primary analysis due to incomplete data collection or excess motion during DXA scanning. To compare our findings with published data from similar trials in the United Kingdom (23) and Denmark (21), pooled analyses of the unadjusted effect estimates for TBLH BMC and aBMD at 3–4 y were completed using a random-effects model with inverse variance weights and restricted maximum likelihood estimation. Heterogeneity was quantified with the *I^2^* statistic (39). Results are expressed as SMDs with 95% CIs. Analyses were conducted using Stata version 15.1 (StataCorp).

## Results

### Participant enrollment and characteristics

A total of 747 MDIG trial participants were identified as potentially eligible for participation in the BONUSKids study; the total proportion of non-enrollment at 4 y (including participants who declined the invitation, were unable to be contacted, or did not meet eligibility criteria) did not differ significantly across the intervention arms (*P* = 0.35 by χ^2^). Eight of the 13 children considered ineligible were excluded due to an inability to schedule a visit within the specified age range (48 ± 3 mo). Of 642 participants who completed the 4-y visit, 608 had a high-quality DXA scan, of whom 599 contributed data to TBLH (93% of total participants enrolled), 565 to WB (88%), and 572 to head-only (89%) measurements. Enrollment was evenly distributed across the 5 trial arms, and participation in study activities, including DXA scanning, was similar by intervention group (**Supplemental Results** and **Supplemental Figure 1**).

Maternal sociodemographic characteristics at randomization were comparable across intervention arms (**Table 1**). In agreement with the MDIG trial findings (26), mean maternal serum 25(OH)D was low (<30 nmol/L) prior to intervention in the present cohort, and increased by delivery in a dose–response manner (Table 1). There were no differences by intervention group in infant characteristics at birth or height and weight at 4 y of age (Table 1). In addition to their greater adherence to the intervention (attributable to the BONUSKids eligibility criteria), mothers of children in BONUSKids were, on average, 1 y older upon MDIG trial enrollment and were more likely to have given birth by cesarean delivery compared with other MDIG participants. There was also a very minor difference in gestational age at enrollment; however, other baseline characteristics were similar between the BONUSKids cohort and the maternal–infant pairs not included in this follow-up study (Supplemental Results and **Supplemental Table 1**).

**FIGURE 1:**
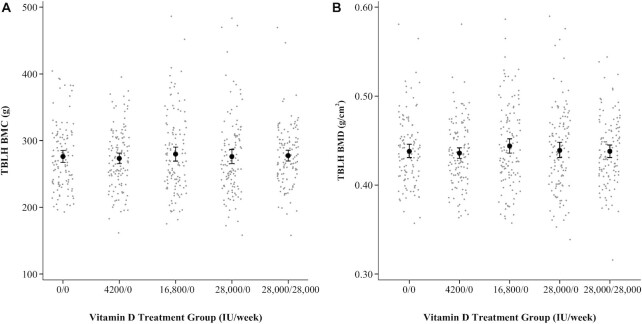
TBLH BMC (A) and aBMD (B) by intervention group (0/0, *n* = 114; 4200/0, *n* = 126; 16,800/0, *n* = 120; 28,000/0, *n* = 121; 28,000/28,000, *n* = 118). Intervention group reflects the vitamin D dose provided in IU/week, represented as a prenatal/postpartum supplementation regimen assigned to the child's mother from randomization (17–24 weeks’ gestation) to 6 mo postpartum. Means and 95% CIs for each intervention group are represented by the solid black circles and vertical lines. aBMD, areal bone mineral density; BMC, bone mineral content; BMD, bone mineral density; TBLH, total-body-less-head.

**TABLE 1:** Maternal and child characteristics of participants in the MDIG trial and BONUSKids follow-up study, by vitamin D intervention group^1^

	Vitamin D (prenatal/postpartum IU/wk)					
	0/0 (*n* = 121)	4200/0 (*n* = 137)	16,800/0 (*n* = 130)	28,000/0 (*n* = 129)	28,000/28,000 (*n* = 125)	*P* ^2^
Maternal characteristics						
Age at enrollment, y						
Median	23	23	23	23	24	0.31
Range	18–38	18–40	18–35	18–35	18–38	
Height at enrollment, cm	150.6 ± 5.8^3^	150.9 ± 5.2	150.3 ± 5.2	150.1 ± 5.5	151.8 ± 5.5	0.12
BMI at enrollment,^4^ kg/m^2^	24.1 ± 4.2	23.1 ± 4.2	23.6 ± 3.7	24.1 ± 3.8	24.5 ± 4.3	0.07
Gestational age at enrollment, wk	20.4 [3.1]^5^	20.3 [3.1]	20.0 [3.3]	20.1 [3.1]	20.1 [3.3]	0.25
Educational level at enrollment, *n* (%)						0.87
Secondary school complete or higher^6^	25/117 (21)	31/131 (24)	26/122 (21)	23/124 (19)	28/122 (23)	
Household asset index quintile at enrollment^7^						0.68
Q1	26/121 (21.5)^8^	28/136 (20.6)	22/130 (16.9)	22/128 (17.2)	19/125 (15.2)	
Q2	22/121 (18.2)	30/136 (22.1)	32/130 (24.6)	22/128 (17.2)	20/125 (16.0)	
Q3	30/121 (24.8)	24/136 (17.7)	22/130 (16.9)	27/128 (21.1)	27/125 (21.6)	
Q4	22/121 (18.2)	23/136 (16.6)	30/130 (23.1)	31/128 (24.2)	33/125 (16.4)	
Q5	21/121 (17.4)	31/136 (22.8)	24/130 (18.5)	26/128 (20.3)	26/125 (20.8)	
Serum 25(OH)D at enrollment,^9^ nmol/L	26.8 ± 14.5	27.5 ± 14.0	28.8 ± 13.4	26.0 ± 12.6	27.3 ± 13.1	0.55
Serum 25(OH)D at delivery,^10^ nmol/L	21.2 ± 11.0^a^	70.2 ± 19.6^a^	97.9 ± 23.3^a^	112.2 ± 26.7^b^	110.0 ± 21.8^b^	<0.001
Hemoglobin at enrollment, g/L	104 ± 12	107 ± 12	106 ± 11	107 ± 10	107 ± 11	0.29
Adherence to prenatal trial supplements, %	99.3 ± 1.9	99.4 ± 2.0	99.5 ± 1.9	99.4 ± 2.4	99.6 ± 1.8	0.87
Child characteristics						
Male sex, *n* (%)	54/121 (44.6)	69/137 (50.4)	71/130 (54.6)	62/129 (48.1)	65/125 (52.0)	0.057
Gestational age at birth, wk	38.7 ± 1.7^a^	39.3 ± 1.3^b^	39.0 ± 1.6^ab^	39.0 ± 1.5^ab^	38.9 ± 1.6^ab^	0.09
Birth weight,^11^ g	2708 ± 355	2732 ± 375	2709 ± 341	2666 ± 312	2733 ± 370	0.70
Mode of delivery, *n* (%)						0.58
Cesarean delivery	62/120 (48)	79/136 (42)	66/130 (49)	66/128 (48)	72/124 (42)	
Vaginal birth	58/120 (52)	57/136 (58)	64/130 (51)	62/128 (52)	52/124 (58)	
Duration of exclusive breastfeeding,^12^ wk	11 [18]	15 [16]	13 [19]	13 [16]	15 [17]	0.41
Height at 4 y,^13^ cm	98.8 ± 4.7	98.6 ± 4.6	98.5 ± 4.3	98.5 ± 4.7	99.1 ± 3.8	0.79
Height-for-age *z* score at 4 y^13^	–1.18 ± 1.08	–1.25 ± 1.04	–1.27 ± 0.98	–1.25 ± 1.07	–1.13 ± 0.90	0.81
Weight at 4 y,^13^ kg	14.4 ± 2.2	14.2 ± 2.0	14.5 ± 2.5	14.3 ± 2.6	14.4 ± 2.3	0.78
Weight-for-age *z* score at 4 y^13^	–1.12 ± 1.12	–1.21 ± 1.04	–1.07 ± 1.20	–1.21 ± 1.24	–1.13 ± 1.05	0.83
BMI-for-age *z* score at 4 y^13^	–0.54 ± 1.00	–0.59 ± 0.93	–0.37 ± 1.12	–0.60 ± 1.34	–0.61 ± 1.05	0.32
Serum 25(OH)D at 4 y,^14^ nmol/L	34.9 ± 13.5	34.1 ± 14.5	36.2 ± 17.1	33.9 ± 16.0	36.0 ± 16.3	0.70

1 Maternal anthropometric, sociodemographic, and biochemical characteristics were recorded upon enrollment (17–24 weeks’ gestation) to the MDIG trial, and therefore reflect characteristics prior to intervention. Maternal data, child characteristics at birth, and breastfeeding patterns were collected during the MDIG trial; child anthropometric and biochemical characteristics at 4 y were collected during the BONUSKids follow-up study. Intervention group reflects the vitamin D dose assigned in IU/week, represented as a prenatal/postpartum dose from enrollment to 6 mo postpartum. BONUSKids, BONe and mUScle Health in Kids; MDIG, Maternal Vitamin D for Infant Growth Trial; 25(OH)D, 25-hydroxyvitamin D.

2 *P* values for differences between groups by ANOVA for normally distributed variables, by Kruskal-Wallis test for non–normally distributed variables, and chi-square test for categorical variables. Values in the same row but with different superscript letters are significantly different (*P* < 0.05 via Tukey's test post ANOVA).

3 Mean ± SD (all such values).

4 Derived from height and weight measurements obtained at 17–24 weeks’ gestation as prepregnancy measurements were not available.

5 Median [IQR] (all such values).

6 Defined as the achievement of a secondary school certification, equivalent to at least 10 y of schooling, at the time of enrollment to the MDIG trial.

7 Determined by claimed ownership of household items, using principal components analysis.

8 Number/total (%) (all such values).

9 *n* in each group: 0/0, 120; 4200/0, 136; 28,000/0, 128; 28,000/28,000, 124 due to missing data.

10 *n* in each group: 0/0, 71; 4200/0, 76; 16,800/0, 84; 28,000/0, 75; 28,000/28,000, 80 due to missing data.

11 Data limited to measurements collected within 48 h of birth. *n*: 0/0 = 89; 4200/0 = 100; 16,800/0 = 98; 28,000/0 = 90; 28,000/28,000 = 88 due to missing data.

12 Defined as the number of continuous weeks from birth in which an infant was classified as exclusively breastfed (received breast milk only), irrespective of breastfeeding practices in the first week of life.

13 *n* in each group: 4200/0, 136 due to 1 missing data; 28,000/28,000, 124 due to 1 missing data.

14 *n* in each group: 0/0, 107; 4200/0, 121; 16,800/0, 123; 28,000/0, 110; 28,000/28,000, 106 due to missing data.

### Effect of maternal vitamin D supplementation on offspring bone mass

In primary unadjusted analyses, there was no difference in TBLH BMC between the prenatal high-dose and placebo groups (**Table 2**). Inferences were unchanged upon adjustment for height, weight, and sex (Supplemental Results and **Supplemental Table 2**). A dose-ranging effect of prenatal supplementation relative to placebo was not evident, nor did we observe a difference in TBLH BMC attributable to continuation of high-dose supplementation throughout lactation (**Figure 1**; **Table 3**; Supplemental Results and **Supplemental Table 3**). CIs for the effect estimates remained wide and inferences unchanged in multivariable analysis adjusting for selected maternal and household characteristics and duration of exclusive breastfeeding (Supplemental Results and **Supplemental Table 4**). Inferences were unchanged using the MICE model (Supplemental Results and **Supplemental Table 5**). Results were also similar using WB and head-only measures (Tables 2 and 3; Supplemental Results and Supplementary Table 3). In line with findings for BMC, there was no effect of high-dose prenatal supplementation compared with placebo on TBLH or WB aBMD in unadjusted analyses (Figure 1; Table 2) or in adjusted analyses accounting for height, weight, and sex (Supplemental Results and Supplemental Table 2). Evidence for a dose-ranging effect across intervention groups was also not present (Figure 1; Table 3). Effects on TBLH aBMD were similar in the MICE model (Supplemental Results and Supplemental Table 5) and multivariable analyses adjusting for selected covariates (Supplemental Results and Supplemental Table 4). Head aBMD was greater in offspring of women who received postpartum supplementation (Table 2), for which the effect was attenuated and no longer significant in height-, weight-, and sex-adjusted analysis (mean difference = 0.019 g/cm^2^; 95% CI: –0.004, 0.041; *P* = 0.11). In sensitivity analyses restricted to DXA scans with no motion artifact, the effect of postpartum supplementation on head aBMD was not evident (Supplemental Results and **Supplemental Table 6**). Visual inspection suggested no association between weekly prenatal supplemental vitamin D intake and BMC or aBMD (Supplemental Results and **Supplemental Figure 2**).

**TABLE 2:** BMC, aBMD, body composition, and grip strength of children at 4 y of age whose mothers were randomly assigned to receive high-dose prenatal vitamin D supplementation (28,000 IU/wk) or placebo from 17–24 weeks’ gestation to delivery^1^

	Prenatal vitamin D					
	0 IU/wk		28,000 IU/wk			
	*n*	Mean ± SD	*n*	Mean ± SD	Difference (95% CI)	*P*
Total-body-less-head						
TBLH BMC, g	114	276.2 ± 48.5	239	276.8 ± 52.8	0.61 (–10.90, 12.13)	0.92
TBLH aBMD, g/cm^2^	114	0.438 ± 0.039	239	0.439 ± 0.043	0.0004 (–0.0089, 0.0097)	0.93
TBLH fat mass, kg	114	3.97 ± 1.17	239	3.94 ± 1.43	–0.03 (–0.33, 0.28)	0.85
TBLH fat tissue mass, %	114	31.7 ± 5.2	239	31.4 ± 5.4	–0.30 (–1.50, 0.89)	0.62
TBLH lean mass, kg	114	8.38 ± 1.20	239	8.34 ± 1.10	–0.04 (–0.29, 0.22)	0.78
Whole-body						
WB BMC, g	109	474.6 ± 65.5	223	481.4 ± 68.4	6.81 (–8.70, 22.32)	0.39
WB aBMD, g/cm^2^	109	0.579 ± 0.045	223	0.584 ± 0.044	0.005 (–0.005, 0.015)	0.32
WB fat mass, kg	109	4.21 ± 1.09	223	4.26 ± 1.39	0.05 (–0.25, 0.35)	0.75
WB fat tissue mass, %	109	30.1 ± 4.6	223	30.0 ± 4.7	–0.09 (–1.15, 0.98)	0.87
WB lean mass, kg	109	9.65 ± 1.22	223	9.70 ± 1.16	0.05 (–0.22, 0.32)	0.71
Head only						
Head BMC, g	110	200.1 ± 24.5	226	201.8 ± 22.2	1.71 (–3.54, 6.96)	0.52
Head aBMD, g/cm^2^	110	1.035 ± 0.095	226	1.054 ± 0.091	0.019 (–0.002, 0.040)	0.08
Functional strength						
Grip strength, kg	120	4.48 ± 1.26	247	4.50 ± 1.33	0.022 (–0.26, 0.31)	0.88

1 Values are means ± SDs unless otherwise indicated. *P* values for difference between groups derived by 2-sided independent-samples *t* test. aBMD, areal bone mineral density; BMC, bone mineral content; TBLH, total-body-less-head; WB, whole-body.

**TABLE 3:** Effect of maternal vitamin D supplementation on offspring BMC, aBMD, and grip strength at age 4 y in all maternal vitamin D intervention groups relative to placebo^1^

		Vitamin D (prenatal/postpartum IU/wk)				
		Mean (95% CI)	Mean difference (95% CI)^2^			
	*n*	0/0	4200/0	16,800/0	28,000/0	28,000/28,000
TBLH BMC,^3^ g	599	276.2 (267.2, 285.2)	–2.9 (–15.0, 9.19)	3.6 (–9.7, 16.9)	–0.1 (–13.6, 13.4)	1.3 (–11.0, 13.6)
TBLH aBMD,^3^ g/cm^2^	599	0.438 (0.431, 0.445)	–0.002 (–0.013, 0.007)	0.006 (–0.005, 0.016)	0.0009 (–0.010, 0.012)	–0.00005 (–0.010, 0.010)
WB BMC,^4^ g	565	474.6 (462.2, 487.0)	–0.96 (–16.0, 14.1)	7.2 (–10.8, 25.1)	3.8 (–15.4, 23.0)	9.9 (–6.1, 26.0)
WB aBMD,^4^ g/cm^2^	565	0.579 (0.570, 0.587)	0.0008 (–0.010, 0.012)	0.010 (–0.002, 0.022)	0.002 (–0.012, 0.015)	0.007 (–0.005, 0.018)
Head BMC,^5^ g	572	200.1 (195.5, 204.7)	–0.9 (–6.6, 4.8)	2.1 (–3.7, 7.9)	–0.5 (–7.2, 6.2)	4.0 (–1.6, 9.6)
Head aBMD,^5^ g/cm^2^	572	1.035 (1.017, 1.053)	0.005 (–0.018, 0.027)	0.017 (–0.006, 0.041)	0.014 (–0.013, 0.040)	0.024* (0.0009, 0.047)
Grip strength,^6^ kg	630	4.48 (4.25, 4.71)	0.004 (–0.31, 0.32)	0.13 (–0.23, 0.49)	–0.002 (–0.33, 0.33)	0.05 (–0.26, 0.35)

1 Effect estimates for between-group differences calculated from unadjusted linear regression models, with placebo (0/0 IU/wk) as the reference group, whereby intervention group reflects the vitamin D dose provided in IU/week, represented as a prenatal/postpartum supplementation regimen assigned to the child's mother from randomization (17–24 weeks’ gestation) to 6 mo postpartum. Estimates of the 95% CIs were obtained using a bootstrap procedure with 1000 replications. *Indicates significant difference relative to placebo at *P* < 0.05. aBMD, areal bone mineral density; BMC, bone mineral content; TBLH, total-body-less-head; WB, whole-body.

2 Values represent mean difference for each vitamin D supplementation group compared with placebo.

3 *n* in each group: 0/0, 114; 4200/0, 126; 16,800/0, 120; 28,000/0, 121; 28,000/28,000, 118.

4 *n* in each group: 0/0, 109; 4200/0, 120; 16,800/0, 113; 28,000/0, 114; 28,000/28,000, 109.

5 *n* in each group: 0/0, 110; 4200/0, 120; 16,800/0, 116; 28,000/0, 115; 28,000/28,000, 111.

6 *n* in each group: 0/0, 120; 4200/0, 134; 16,800/0, 129; 28,000/0, 125; 28,000/28,000, 122.

### Effect of maternal vitamin D supplementation on grip strength and body composition

A dose-ranging effect of maternal supplementation on maximum grip strength at 4 y of age was not evident, nor did we observe a specific effect attributable to postpartum vitamin D supplementation (Table 2; Table 3). Findings were unchanged in multivariable analysis (Supplemental Results and Supplemental Table 4), following application of MICE to account for missing data (Supplemental Results and Supplemental Table 5), and when using mean grip strength as the outcome (*P* > 0.05; data not shown). TBLH and WB fat mass (kilograms and %) and lean mass were similar across all intervention groups at 4 y of age (Supplemental Results and Supplemental Table 3). High-dose prenatal supplementation did not affect WB body composition relative to placebo in either unadjusted or adjusted analyses (Supplemental Results and Supplemental Table 2). No difference in TBLH or WB FFM or FMI was found in each intervention group compared with placebo (*P* > 0.05 for all intervention groups across each outcome; data not shown).

Findings for all outcomes were similar after excluding DXA scans with minor motion (Supplemental Results and Supplemental Table 6).

### Subgroup analyses by child sex

Relative to placebo, there were no between-group differences in TBLH BMC between boys, in contrast to the higher mean TBLH BMC in girls of women who received postpartum supplementation (28,000 IU/wk) (Supplemental Results and **Supplemental Table 7**). The effect in girls was substantially attenuated and no longer statistically significant after adjustment for height and weight at 4 y of age (mean difference = 2.9 g; 95% CI = –3.99, 9.77; *P* = 0.41). Between-group differences in child height were not observed at 4 y in unstratified analyses involving both boys and girls (Table 1); however, the sex-specific effect on TBLH BMC was attributable to a greater mean height of girls in the 28,000/28,000-IU/wk trial arm relative to placebo (mean difference = 1.64 cm; 95% CI: 0.13, 3.16; *P* = 0.034), despite an absence of an effect of maternal vitamin D supplementation on linear growth in girls up to 2 y of age (Supplemental Results and **Supplemental Table 8**).

As with TBLH BMC, a similar sex-specific effect was observed for TBLH aBMD; the sex-by-intervention group interaction term for the 28,000/28,000-IU/wk regimen was significant (Supplemental Results and Supplemental Table 7), and was attenuated upon adjustment for child height and weight (mean difference = 0.008 g/cm^2^; 95% CI = –0.006, 0.023; *P* = 0.23). The lack of effect of vitamin D on grip strength, fat mass (kilograms and %), and lean mass was consistent in analyses stratified by sex (Supplemental Results and Supplemental Table 7).

### Subgroup analyses by maternal vitamin D status at enrollment

There was no interaction of maternal baseline 25(OH)D with intervention group for any outcome (*P* > 0.05 for all intervention groups across each outcome; data not shown). Analysis restricted to offspring of women with vitamin D deficiency at enrollment also showed no effect of vitamin D on TBLH BMC (Supplemental Results and **Supplemental Table 9**). In contrast to primary analyses, the significant difference in head aBMD between the postpartum supplemented group compared with placebo was not observed when restricted to women with vitamin D deficiency (Supplemental Results and Supplemental Table 9).

### Analyses restricted to term-born infants

Inferences from primary analyses for all outcomes were similar when restricted to term-born infants (Supplemental Results and **Supplemental Table 10**). The greater head-only aBMD of the 28,000/28,000-IU/wk group in primary analyses was also present when restricted to term infants (Supplemental Results and Supplemental Table 10), and followed similar attenuation upon adjustment for height, weight, and sex (mean difference vs. placebo = 0.020 g/cm^2^; 95% CI: –0.003, 0.043; *P* = 0.09).

### Meta-analysis of 3 prenatal vitamin D supplementation trials

Pooled analysis did not support an effect of prenatal vitamin D supplementation on offspring TBLH BMC at 3–4 y of age; specifically, the pooled SMD was of small magnitude and not statistically significant (SMD = 0.077; 95% CI: –0.047, 0.201; *P* = 0.22 for effect estimate; *I*^2^ = 0.0%) (Supplemental Results and **Supplemental Table 11**). Similar findings were observed for TBLH aBMD, for which the SMD of each study showed either null or small positive effects of the intervention relative to placebo, and no overall effect in pooled analysis (SMD = 0.098; 95% CI: –0.037, 0.233; *P* = 0.15 for effect estimate; *I*^2^ = 13.8%) (Supplemental Results and Supplemental Table 11).

## Discussion

In a placebo-controlled, dose-ranging trial, maternal prenatal with or without postpartum vitamin D supplementation did not increase early childhood BMC, aBMD, or grip strength. Effects on fat and lean mass were also not observed. While previous trials have shown mixed results among populations who were relatively vitamin D replete (21–23), the present findings demonstrate a lack of benefit in a population in which a majority of pregnant women had low 25(OH)D at randomization, despite elimination of biochemical vitamin D deficiency with high-dose vitamin D supplementation.

While sex-specific differences in body composition and growth trajectories have been well established (29, 30, 40), we are not aware of evidence substantiating a sex-specific mechanism by which vitamin D would influence skeletal deposition in childhood. Prespecified secondary analyses revealed a significant interaction with sex, such that there was a potential benefit of combined prenatal and postpartum vitamin D on bone mineral accrual in girls; however, the effect was explained by differences in body size as girls in this group had a greater mean height compared with the placebo group. Given that this height difference was absent at 12 and 24 mo, the difference in height and corresponding greater BMC at 4 y of age was most likely a chance finding in the context of multiple comparisons, and unlikely to represent a causal effect of the vitamin D intervention in girls specifically. Although it remains possible that an early-life sex-specific programming effect of vitamin D in our cohort manifested after 2 y of age, further exploration of sex-specific effects in future trials are required to corroborate these findings.

In contrast to the null effects on TBLH and WB aBMD, head aBMD was significantly greater in offspring of women who received high-dose prenatal and postpartum supplementation. Given the large contribution of the skull to WB BMC in young children, and because movement of the head region is a common cause of DXA artifacts (32), head-only measures were not used as primary outcomes. The effect on head aBMD was nonsignificant in subgroup analyses by sex or upon exclusion of DXA scans with minor motion artifact, and multiple comparisons may have led to a false-positive result. Yet, the difference in head aBMD between the 28,000/28,000-IU/wk and placebo groups was of moderate magnitude (SMD = 0.3), and the finding agreed with that of the Copenhagen Prospective Studies on Asthma in Childhood (COPSAC) trial (21). Craniotabes is a clinical feature of vitamin D–deficiency rickets in young infants (41), and murine models suggest that 1,25-dihydroxyvitamin D may regulate bone formation differently via local effects on flat bones such as the skull (intramembranous ossification) compared with long bones (endochondral ossification) (42). In separate analyses of the MDIG trial cohort, infants in the 28,000/28,000-IU/wk group had substantially higher 25(OH)D until 6 mo of age (26), and a lower risk of biochemical rickets (43). It is plausible that improved vitamin D status in early infancy led to increased cranial mineralization due to local bone-specific effects of vitamin D rather than global effects on WB mineralization as a result of effects of vitamin D on intestinal calcium absorption.

Effect estimates for primary analyses of the TBLH outcomes had wide CIs, which, based on a priori power calculations, were not a result of limited precision to detect meaningful between-group differences in BMC and aBMD. SDs relative to the population mean for BMC and aBMD were of similar magnitude, as previously reported in this age group (21, 23, 28). Moreover, the magnitudes of differences between groups were small compared with the SDs; even if the observed effects had been statistically significant, they would likely be too small to be clinically important with respect to lowering fracture risk of the long bones (1). Pooling the present results with 2 trials in high-income countries demonstrated a lack of evidence in support of a beneficial effect of prenatal vitamin D on offspring bone mass, but also highlighted heterogeneity in effect sizes across trials and, hence, the need for cautious interpretation of pooled estimates. A recent report from the Maternal Vitamin D Osteoporosis Study (MAVIDOS) trial suggests a positive effect of in utero vitamin D exposure on TBLH aBMD in later childhood (23), despite no effect on WB neonatal bone mass or density (22). Similarly, effects of prenatal vitamin D in the COPSAC trial were inconsistent across TBLH, WB, and head-only outcomes at the 3- and 6-y time points (21). Benefits reported in the COPSAC study were generally only statistically significant in analyses that adjusted for weight, height, and age, yet we confirmed that our conclusion of a null intervention effect was unaffected by covariate adjustment. Nonetheless, we acknowledge the possibility of between-population variations in the effects of early-life vitamin D exposures on bone outcomes. Using published age-specific reference equations from the United Kingdom (30), low mean BMC *z* scores in the present study suggested an overall delay in bone mineral accrual, as expected given the children's relatively short stature. Dietary deficits in calcium or other nutrients involved in bone formation (e.g., essential amino acids) may have been rate-limiting and attenuated potential benefits from maternal vitamin D supplementation in this setting. Furthermore, vitamin D status of the children at 4 y was relatively low across all groups, which may be partly explained by the limited availability of vitamin D–fortified foods (44). Therefore, effects of increased 25(OH)D in infancy may not have been sustained in the context of relative vitamin D deficits throughout the preschool period.

Beyond its role in bone mineral metabolism, vitamin D has been shown to regulate muscle cell proliferation and differentiation, muscle contractility, and adipocyte function (45, 46). However, the limited trial evidence does not support a direct effect of infant vitamin D supplementation on adiposity in early childhood (47), and the present findings mirror those of previous maternal vitamin D trials showing no effect of prenatal vitamin D supplementation on neonatal (22), infant (24), or early childhood (21) lean and fat mass. The Southampton Women's Survey showed an association between maternal late-gestation 25(OH)D and child grip strength (25), but neither the MAVIDOS trial (23) nor the present trial corroborated such an effect using a randomized design.

Several limitations of the study should be acknowledged. Calcium supplementation was provided to all women to mitigate effects of the habitually low dietary calcium intakes that are common in Bangladesh, yet this co-intervention may have compensated for the relative vitamin D deficiency of women in the control group and therefore attenuated intervention effects (48, 49). Supplementation began in the second trimester, and thus coincided with the period in which the majority of maternal–fetal calcium transfer and fetal bone mineral deposition occurs (37). Since DXA-derived measures of neonatal bone mass were not obtained, it remains possible that early differences were present but attenuated over time. Yet, in contrast to the MAVIDOS trial (22, 23), the present findings argue against a latent early-programming effect of vitamin D on offspring bone. Children's diets (50, 51) and physical activity levels (52) may be important determinants of skeletal health and motor development. Although analyses of dietary intake or physical activity were not included in the present study, we expected similar distributions of these factors across intervention groups based on the randomized design. In order to leverage advantages of the MDIG randomized controlled trial design, enrollment in the present study was limited to a subset of MDIG participants who were willing and available to participate in follow-up procedures; therefore, we cannot confirm generalizability of the present findings to the original cohort or the broader population of children in Dhaka and similar settings. Last, DXA is a valid and widely used method for measurement of bone mass; however, further exploration of bone integrity by peripheral quantitative computed tomography may provide an understanding of the effect of vitamin D on skeletal microstructure and, hence, resistance to fractures.

In conclusion, in a population with a high prevalence of vitamin D deficiency, maternal prenatal, with or without postpartum, vitamin D supplementation did not increase child BMC, aBMD, or grip strength at 4 y of age, nor were there associated effects on fat or lean mass. These findings do not support the use of routine prenatal or postpartum maternal vitamin D supplementation for improvement in musculoskeletal health in early childhood.

## Supplementary Material

nqab396_Supplemental_File

## Data Availability

Data described in the manuscript, code book, and analytic code will be made available upon request to the authors. De-identified individual participant data will be provided for use in secondary data analyses approved by an independent research ethics board, and data requestors will be required to sign a data access agreement.

## Notes

This research was supported by funding from the Canadian Institutes for Health Research (PJT159596) and the Bill and Melinda Gates Foundation (OPP1066764). Under the grant conditions of the Bill and Melinda Gates Foundation, a Creative Commons Attribution 4.0 Generic License has already been assigned to the Author Accepted Manuscript version that might arise from this submission. The funding agencies were not involved in the design, implementation, analysis, or interpretation of the data.

Supplemental Methods, Supplemental Results, Supplemental Figures 1 and 2, and Supplemental Tables 1–11 are available from the “Supplementary data” link in the online posting of the article and from the same link in the online table of contents at https://academic.oup.com/ajcn/.

Abbreviations used: aBMD, areal bone mineral density, BMC, bone mineral content; BMD, bone mineral density; BONUSKids, “BONe and mUScle health in Kids” study; COPSAC, Copenhagen Prospective Studies on Asthma in Childhood; FFM, fat-free mass; FMI, fat mass index; LLoQ, lower limit of quantification; MAVIDOS, Maternal Vitamin D Osteoporosis Study; MDIG, Maternal Vitamin D for Infant Growth; MICE, multiple imputation by chained equations; SMD, standardized mean difference; TBLH, total-body-less-head; WB, whole-body; 25(OH)D, 25-hydroxyvitamin D; 25(OH)D_2_, 25-hydroxyvitamin D_2_; 25(OH)D_3_, 25-hydroxyvitamin D_3_; 3-epi-25(OH)D_3_, 3-epi-25-hydroxyvitamin D_3_.
