# Supplementary material for: Effect of maternal prenatal and postpartum vitamin D supplementation on offspring bone mass and muscle strength in early childhood: follow-up of a randomized controlled trial
Source: Am J Clin Nutr. 2021 Nov 27;115(3):770–80. doi: 10.1093/ajcn/nqab396 (PMC8895216; doi:10.1093/ajcn/nqab396)
Supplement: nqab396_Supplemental_File [file nqab396_supplemental_file.pdf]

Effect of maternal prenatal and postpartum vitamin D supplementation on offspring bone mass and muscle strength in early childhood: follow-up of a randomized controlled trial

O'Callaghan KM et al.

Online Supplementary Material

Authors: Karen M O' Callaghan, Shaila S Shanta, Farzana Fariha, Jennifer Harrington, Abdullah Al Mahmud, Abby L Emdin, Alison D Gernand, Tahmeed Ahmed, Steven A Abrams, Daniel R Moore and Daniel E Roth.

Address correspondence to: Daniel E. Roth, Department of Paediatrics, Hospital for Sick Children and University of Toronto, 686 Bay Street, Toronto ON, M5G 0A4, Canada; +1 416-813-7654 ext. 328807; daniel.roth@sickkids.ca

Table of Contents

Supplementary Methods 4

Sample size estimation, calculation of anticipated detectable differences, and power calculation for secondary outcomes 4

Criteria for appraisal of DXA scan images for estimates of bone and body composition 6

Supplementary Results 8

Supplementary Table 1: Comparison of maternal and child characteristics of MDIG trial participants who participated versus those who did not participate in the BONUSKids follow-up study 8

Supplementary Table 2: Comparison of mean difference in offspring bone mineral content, bone mineral density, and body composition of maternal high-dose vitamin D supplementation versus placebo in two trials 10

Supplementary Table 3: Bone mineral content, bone mineral density, body composition and grip strength at 4 years of age, by intervention group 12

Supplementary Table 4: Effect of maternal vitamin D supplementation on offspring total-body-less-head bone mineral content, total-body-less-head bone mineral density and grip strength at age four years in all maternal vitamin D intervention groups relative to placebo, adjusting for covariates 14

Supplementary Table 5: Effect of maternal vitamin D supplementation on offspring total-body-less-head bone mineral content, total-body-less-head bone mineral density and grip strength at age four years in all maternal vitamin D intervention groups relative to placebo, using multiple imputation by chained equations to address missing values 15

Supplementary Table 6: Effect of maternal vitamin D supplementation on offspring bone mineral content and bone mineral density at age four years relative to placebo among participants whose DXA report showed only negligible or no motion artifact 16

Supplementary Table 7: Effect of maternal vitamin D supplementation on offspring bone mineral content, bone mineral density and grip strength at age four years relative to placebo, by sex 18

Supplementary Table 8: Length- and height-for-age z-scores of girls from birth to 4 years of age, limited to placebo and high-dose prenatal vitamin D supplementation trial arms and stratified by study cohort 21

Supplementary Table 9: Effect of maternal vitamin D supplementation on offspring bone mineral content, bone mineral density and grip strength at age four years relative to placebo among offspring of women who were vitamin D deficient at randomization 23

Supplementary Table 10: Effect of maternal vitamin D supplementation on offspring bone mineral content, bone mineral density and grip strength at age four years relative to placebo and restricted to term-born infants only 25

Supplementary Table 11: Pooled analysis showing standardized mean differences for offspring total-body-less head bone mineral content and areal density at 3-4 years of age in response to prenatal vitamin D supplementation compared to placebo 27

Supplementary Figure 1: CONSORT Flow Diagram of participant enrolment, random assignment and participation in study activities throughout the study by intervention group 28

Supplementary Figure 2: Bivariate relationships between (A) BMC and prenatal supplemental vitamin D intake (IU/week) and (B) aBMD and prenatal supplemental vitamin D intake (IU/week) 30

Supplementary References........ 31

Supplementary Methods

Sample size estimation, calculation of anticipated detectable differences, and power calculation for secondary outcomes

The target sample size was based on the primary objective of detecting a meaningful difference in total-body-less-head (TBLH) bone mineral content (BMC) at 4 years of age between the placebo and combined high-dose (28000 IU/week prenatal with or without 28000 IU/week postpartum) groups. However, we also performed power calculations for selected secondary outcomes. Including allowance for an expected attrition rate of 15%, the desired sample size for our primary outcome was calculated at 140 children from each of the 5 groups in the original MDIG trial, giving an overall target sample size of 700 children. Based on the MDIG participant follow-up registry, we anticipated that about half of the original MDIG trial sample (1298 pregnancies) would be available and willing to consider participation in a follow-up study (i.e., ~650 to 700 participants). Therefore, we aimed to enrol the maximal number of children available from the original cohort, and expected an even distributed across trial arms given the randomized trial design.

For lumbar spine areal BMD (aBMD), we estimated that a SMD=0.36 would be an approximate difference of 0.02 g/cm^2 or 4.8% assuming a mean of 0.46 g/cm^2 and CV of 13% (1). A separate originally planned analysis of lumbar spine aBMD was determined to be unfeasible given the additional time and cost of conducting the DXA scanning in a manner that would generate the necessary data.

In extension of the between-group differences in our primary analytical approach, secondary analyses were based on the dose-response relationship using supplemental vitamin D intake as the continuous exposure variable. Assuming a 5% risk of a type I error and *n*=600 (where *n*=120 from each trial arm), we anticipated having 90% power to detect a minimum SMD=0.12 for every 10,000 IU/week increase in vitamin D intake. Previous research suggested this is a plausible dose-response effect (assuming 10,000 IU/week yields a ~25 nmol/L difference in 25(OH)D): 0.24 SMD increase in BMC at 20 years of age for each 25 nmol/L increase in maternal 25(OH)D was found in an Australian cohort (2), equating to a ~8 g increase in BMC per 10,000 IU/week.

For analyses of lean and fat mass, we considered that we would have 90% power to detect differences of 1.7, 1.5 and 0.6 percentage points, respectively, given distributions at age 4 years (3). In the dose-response analysis based on vitamin D dose as a continuous variable, we expected to be able to detect 0.5 %-point increases in lean and fat mass.

Criteria for appraisal of DXA scan images for estimates of bone and body composition.

**Classification**	**Criteria**	**Primary analytical approach**	**Sensitivity analysis (more stringent criteria)**
	**Assessment of motion artifact**		
No (or negligible) motion artifact	• No (or negligible) motion artifact<br>• Whole body positioned within identified measurement parameters<br>• No presence of additional artifacts within the measurement area (e.g. hand of caregiver/DXA technician)	Include	Include
Minor motion artifact	• Small motion artifact not considered to affect results (e.g. slight movement of one limb)<br>• Peripheral body parts are outside the measurement parameter (including hands and feet) but the DXA software has provided an estimated value based on the other (non-missing) side of the body (4)<br>• Image suggests the participant was inappropriately dressed, including metal accessories (e.g. small button on jeans) that would not be considered to affect results	Include	Exclude
Major motion artifact	• Obvious motion artifact from the base of the neck downwards such that the scan is uninterpretable, according to published recommendations (5)<br>• Peripheral body parts are well outside the measurement parameter (including hands and feet) such that DXA software did not provide an estimated value based on the other (non-missing) side of the body (4)<br>• Image suggests the participant was inappropriately dressed, including obvious large metal accessories (e.g. zip and/or buttons on jeans) such that a BMC value for that area cannot be obtained<br>• Presence of additional artifacts within the measurement area (e.g. hand of caregiver/DXA technician)	Exclude	Exclude

Classification	Criteria	Primary analytical approach	Sensitivity analysis (more stringent criteria)
	**Assessment of head alignment**		
Acceptable head orientation	• The whole head is positioned within the measurement area (ROI), and is positioned with the head facing upward, perpendicular to the DXA bed surface or at an angle of <45 degrees<br>• The head is not tilted forward such that the ROI for the head is easily distinguished from the chest and shoulders	Include	Include
Unacceptable head orientation	• Only part of the head is positioned within the measurement area (ROI) or head is turned outward (i.e., not face-up and perpendicular to the DXA bed surface) to an angle of ≥45 degrees<br>• The head is tilted too far forward such that only part of the head is included in the measurement area (less than 80%; typically excluding the area from the mouth downwards) or the full shoulders (i.e., beyond the upper tip of the deltoid) are included in the measurement for the head region	Exclude	Exclude

Supplementary Results

Supplementary Table 1: Comparison of maternal and child characteristics of MDIG trial participants who participated versus those who did not participate in the BONUSKids follow-up study[1,2].

	Did not participate in BONUSKids (*n*=656)	Participated in BONUSKids (*n*=642)	***P***
Maternal characteristics			
Age at enrolment, years			
Median	22	23	0.004
Range	18-39	18-40	
Height at enrolment[3], cm	151.1 ± 5.3	150.7 ± 5.5	0.18
BMI at enrolment[4], kg/m²	23.5 ± 4.0	23.9 ± 4.1	0.08
Gestational age at enrolment, weeks			
Median	20.4	20.3	0.034
Range	17-24	17-24	
Education level, *n* (%)			
Secondary school complete or higher[5]	152 (23)	134 (21)	0.32
Household asset index quintile[6,7]			
Q1	144 (22.5)	117 (18.3)	
Q2	125 (19.5)	126 (19.7)	
Q3	126 (19.7)	130 (20.3)	0.33
Q4	118 (18.4)	139 (21.7)	
Q5	127 (19.8)	128 (20.0)	
Serum 25(OH)D[8], nmol/L	27.6 ± 14.5	27.3 ± 13.5	0.7
Hemoglobin, g/L	106.0 ± 11.5	106.3 ± 11.3	0.6
Adherence with prenatal trial supplements,%	92 ± 21	99 ± 2	<0.001

	Did not participate in BONUSKids (*n*=656)	Participated in BONUSKids (*n*=642)	*P*
Child characteristics			
Male sex, *n* (%)	52	50	0.5
Gestational age at birth[9], weeks	32 ± 2	39 ± 2	0.7
Birthweight[10], g	2721 ± 367	2710 ± 351	0.6
Mode of delivery, *n* (%)			
C-section	308 (48)	345 (54)	0.038
Vaginal birth	330 (52)	293 (46)	

[1]Maternal anthropometric, sociodemographic and biochemical characteristics recorded upon enrolment to the Maternal Vitamin D for Infant Growth Trial (MDIG), and therefore reflect characteristics prior to intervention. *P* values for differences between groups by independent samples *t*-tests and Wilcoxon rank-sum tests for normally and non-normally distributed continuous data, respectively, and by chi-square (χ^2) tests for comparison categorical data. 25(OH)D, 25-hydroxyvitamin D; BONUSKids, BONe and mUScle health in Kids study; MDIG, Maternal Vitamin D for Infant Growth trial.

[2]Inferences were similar in additional analyses of within-group comparisons in which baseline characteristics of each intervention group of the MDIG only cohort was compared to the characteristics of the same intervention group in the MDIG and BONUSKids cohort.

[3]Data are presented as mean ± SD (all such values).

[4]Derived from height and weight measures taken at 17-24 weeks of gestation as pre-pregnancy measures were not available.

[5]Defined as the achievement of a secondary school certification, equivalent to at least 10 years of schooling, at the time of enrolment to the MDIG trial.

[6]Data presented as number/total (%) (all such values).

[7]Determined by ownership of household items, using principal components analysis.

[8]*n*=638 for BONUSKids; *n*=653 for MDIG.

[9]*n*=612 for MDIG.

[10]Data limited to measurements collected within 48 hours of birth. *n*=465 for BONUSKids; *n*=370 for MDIG.

Supplementary Table 2: Comparison of mean difference in offspring bone mineral content, bone mineral density, and body composition of maternal high-dose vitamin D supplementation versus placebo in two trials[1].

	COPSAC Aged 3 years[2]				COPSAC Aged 6 years[3]				BONUSKids Ages 4 years[4]			
	Unadjusted mean difference	*P*	Adjusted mean difference[5]	*P*	Unadjusted mean difference	*P*	Adjusted mean difference[2]	*P*	Unadjusted mean difference	*P*	Adjusted mean difference[6]	*P*
TBLH BMC, g	5.00	0.41	6.1	0.05	8.4	0.33	7.8	0.03	0.61	0.92	0.837	0.72
WB BMC, g	12.7	0.16	9.9	0.04	15.4	0.15	13.9	0.01	6.81	0.39	1.04	0.78
Head BMC, g	2.50	0.46	-0.6	0.82	6.3	0.06	6.1	0.03	1.71	0.52	-0.317	0.90
TBLH aBMD, g/cm^2	0.0000	1.0	0.005	0.14	0.01	0.031	0.005	0.15	0.0004	0.93	0.00078	0.81
WB aBMD, g/cm^2	0.01	0.10	0.007	0.16	0.01	0.051	0.009	0.04	0.005	0.32	0.0029	0.52
Head aBMD, g/cm^2	0.02	0.14	0.012	0.34	0.03	0.015	0.033	0.01	0.019	0.082	0.0140	0.18
WB fat mass, g	-40	0.73	71.2	0.50	-175	0.23	-105.2	0.40	48.78	0.75	24.58	0.84
WB lean mass, g	104	0.57	76.7	0.55	110	0.59	-10.7	0.92	52.12	0.71	-78.17	0.30

[1]Mean differences represented by comparison of high dose vitamin D supplementation group versus placebo. *P* value for unadjusted estimates by independent samples *t*-test and adjusted estimates by multiple linear regression. *P* value for unadjusted estimates in the COPSAC study are calculated values derived from the reported means and SDs, and were not provided by the authors in the original publication (6). aBMD; areal

bone mineral density; BMC, bone mineral content; BONUSKids, BONe and mUScle health in Kids study; COPSAC, Copenhagen Prospective Studies on Asthma in Childhood; TBLH, total-body-less-head; WB, whole-body.

[2]N for TBLH = 94 receiving vitamin D and 105 receiving placebo; N Head = 105 receiving vitamin D and 113 receiving placebo; N WB = 82 receiving vitamin D and 95 receiving placebo.

[3]N for TBLH = 187 receiving vitamin D and 196 receiving placebo; N Head = 187 receiving vitamin D and 196 receiving placebo; N WB = 187 receiving vitamin D and 196 receiving placebo.

[4]N for TBLH = 239 receiving vitamin D and 114 receiving placebo; N Head = 226 receiving vitamin D and 110 receiving placebo; N WB = 223 receiving vitamin D and 109 receiving placebo.

[5]BMC and aBMD adjusted for age, sex, height and weight (all such values); fat and lean mass adjusted for age, sex, height and height^2.

[6]BMC and aBMD adjusted for sex, height and weight (all such values); fat and lean mass adjusted for sex, height and height^2. Bootstrapping was applied to all adjusted models (1000 replications).

Supplementary Table 3: Bone mineral content, bone mineral density, body composition and grip strength at 4 years of age, by intervention group[1].

	Vitamin D (prenatal; postpartum IU/week)				
	0;0	4200;0	16800;0	28000;0	28000;28000
**Total-body-less-head[2]**					
TBLH BMC, g	276.2 ± 48.5	273.3 ± 44.9	279.8 ± 58.6	276.1 ± 59.8	277.5 ± 44.8
TBLH aBMD, g/cm^2	0.438 ± 0.039	0.436 ± 0.035	0.444 ± 0.046	0.439 ± 0.047	0.438 ± 0.038
TBLH fat mass, kg	3.97 ± 1.17	3.84 ± 1.03	4.04 ± 1.45	4.01 ± 1.53	3.87 ± 1.31
TBLH fat tissue mass, %	31.7 ± 5.2	31.2 ± 4.6	31.5 ± 5.8	31.9 ± 5.7	31.0 ± 5.1
TBLH lean mass, kg	8.38 ± 1.20	8.32 ± 1.11	8.50 ± 1.26	8.27 ± 1.21	8.41 ± 0.99
TBLH BMC Z-score[3]	-0.95 ± 0.88	-0.96 ± 0.92	-0.93 ± 1.00	-0.89 ± 1.12	-0.92 ± 0.92
TBLH aBMD Z-score[3]	-1.38 ± 0.97	-1.47 ± 0.89	-1.28 ± 1.13	-1.38 ± 1.17	-1.41 ± 0.98
**Whole-body[4]**					
WB BMC, g	474.6 ± 65.5	473.6 ± 52.7	481.8 ± 73.7	478.4 ± 79.7	484.5 ± 54.4
WB aBMD, g/cm^2	0.579 ± 0.045	0.579 ± 0.033	0.588 ± 0.043	0.583 ± 0.049	0.585 ± 0.037
WB fat mass, kg	4.21 ± 1.09	4.13 ± 0.96	4.32 ± 1.42	4.31 ± 1.50	4.21 ± 1.27
WB fat tissue mass, %	30.1 ± 4.4	29.7 ± 3.9	30.0 ± 5.1	30.4 ± 5.0	29.6 ± 4.5
WB lean mass, kg	9.65 ± 1.22	9.66 ± 1.13	9.84 ± 1.27	9.62 ± 1.28	9.78 ± 1.00
**Head only[5]**					
Head BMC, g	200.1 ± 24.5	199.2 ± 19.1	202.2 ± 21.0	199.6 ± 25.5	204.1 ± 18.0
Head aBMD, g/cm^2	1.035 ± 0.095	1.040 ± 0.074	1.053 ± 0.086	1.049 ± 0.102	1.059 ± 0.079

	Vitamin D (prenatal; postpartum IU/week)				
	0;0	4200;0	16800;0	28000;0	28000;28000
Grip strength[5], kg	4.48 ± 1.26	4.48 ± 1.25	4.61 ± 1.55	4.48 ± 1.43	4.53 ± 1.23

[1]All values presented as mean ± SD. aBMD; areal bone mineral density; BMC, bone mineral content; TBLH, total-body-less-head; WB, whole-body.

[2]N 0;0 = 114; 4200;0 = 126; N 16800;0 = 120; 28000;0 = 121; N 28000;28000 = 118.

[3]Calculated using the Lambda-Mu-Sigma (LMS)-modelled formulas derived by Crabtree *et al.* (7).

[4]N 0;0 = 109; 4200;0 = 120; N 16800;0 = 113; 28000;0 = 114; N 28000;28000 = 109.

[5]N 0;0 = 110; 4200;0 = 120; N 16800;0 = 116; 28000;0 = 115; N 28000;28000 = 111.

[6]N 0;0 = 120; 4200;0 = 134; N 16800;0 = 129; 28000;0 = 125; N 28000;28000 = 122.

Supplementary Table 4: Effect of maternal vitamin D supplementation on offspring total-body-less-head bone mineral content, total-body-less-head bone mineral density and grip strength at age four years in all maternal vitamin D intervention groups relative to placebo, adjusting for selected covariates[1].

		Vitamin D (prenatal; postpartum IU/week)				
		0;0	4200;0	16800;0	28000;0	28000;28000
	N	Mean (95% CI)	Mean Difference (95% CI)[2]			
TBLH BMC[3], g	554	276.1 (266.8, 285.4)	-3.1 (-14.5, 8.3)	5.4 (-8.4, 19.2)	-0.7 (-14.0, 12.7)	-3.8 (-15.1, 7.5)
TBLH aBMD[3], g/cm^2	554	0.438 (0.430, 0.445)	-0.002 (-0.011, 0.008)	0.007 (-0.004, 0.018)	0.0004 (-0.011, 0.011)	-0.003 (-0.013, 0.007)
Grip strength[4], kg	585	4.50 (4.27, 4.74)	-0.06 (-0.40, 0.26)	0.14 (-0.20, 0.49)	-0.09 (-0.45, 0.26)	-0.10 (-0.42, 0.23)

[1]Effect estimates for between-group differences calculated from multivariable linear regression models, with placebo (0;0 IU/week) as the reference group whereby intervention group reflects the vitamin D dose provided in IU/week, represented as a prenatal; postpartum supplementation regimen assigned to the child's mother from randomization (17-24 weeks of gestation) to 6 months postpartum. Regression models included the following selected covariates: maternal age at enrolment, maternal height at enrolment, household asset index, and duration of exclusive breastfeeding. Estimates of the 95% CIs were obtained using a bootstrap procedure with 1000 replications. aBMD, areal bone mineral density; BMC, bone mineral content; TBLH, total-body-less-head.

[2]Values represent mean difference for each vitamin D supplementation group compared to placebo.

[3]N 0;0, = 110; 4200;0 = 115; 16800;0 = 114; 28000;0 = 106; 28000;28000 = 109.

[4]N 0;0 = 116; 4200;0 = 123; 16800;0 = 123; 28000;0 = 110; 28000;28000 = 113.

Supplementary Table 5: Effect of maternal vitamin D supplementation on offspring total-body-less-head bone mineral content, total-body-less-head bone mineral density and grip strength at age four years in all maternal vitamin D intervention groups relative to placebo, using multiple imputation by chained equations to address missing values[1].

		**Vitamin D (prenatal; postpartum IU/week)**			
		**4200;0**	**16800;0**	**28000;0**	**28000;28000**
	**N**	**Mean Difference (95% CI)[2]**			
TBLH BMC, g	642	-2.3 (-15.3, 10.6)	4.1 (-9.0, 17.2)	-1.2 (-14.3, 11.9)	-2.3 (-11.0, 15.5)
TBLH aBMD, g/cm^2	642	-0.002 (-0.012, 0.008)	0.006 (-0.004, 0.017)	0.0004 (-0.010, 0.011)	0.0007 (-0.010, 0.011)
Grip strength, kg	642	-0.02 (-0.35, 0.32)	0.13 (-0.21, 0.47)	-0.01 (-0.35, 0.32)	0.04 (-0.30, 0.39)

[1]Effect estimates for between-group differences calculated from linear regression models, with placebo (0;0 IU/week) as the reference group whereby intervention group reflects the vitamin D dose provided in IU/week, represented as a prenatal; postpartum supplementation regimen assigned to the child's mother from randomization (17-24 weeks of gestation) to 6 months postpartum. The imputation models included the main exposure (treatment assignment), the outcome of interest, and auxiliary variables that helped improve the performance of the missing data procedure, including maternal age at enrolment, maternal height at enrolment, household asset index at enrolment, paternal education at enrolment, maternal adherence to the intervention, gestational age at birth, child sex, feeding pattern at 6 months, child anthropometry at 4 years of age, child hemoglobin concentration at 4 years of age, information related to venous blood collection at 4 years of age (successful/not successful), and household smoking status at 4 years of age. aBMD, areal bone mineral density; BMC, bone mineral content; TBLH, total-body-less-head.

[2]Values represent mean difference for each vitamin D supplementation group compared to placebo.

Supplementary Table 6: Effect of maternal vitamin D supplementation on offspring bone mineral content and bone mineral density at age four years relative to placebo among participants whose DXA report showed only negligible or no motion artifact[1].

	Vitamin D (prenatal; postpartum IU/week)				
	**0;0**	**4200;0**	**16800;0**	**28000;0**	**28000;28000**
	**Mean (95% CI)**	**Mean Difference (95% CI)[2]**			
TBLH BMC[3], g	272.1 (262.6, 281.6)	1.4 (-10.5, 13.3)	6.8 (-7.4, 21.1)	4.8 (-9.4, 19.0)	3.5 (-8.3, 15.4)
TBLH aBMD[3], g/cm^2	0.434 (0.427, 0.442)	0.001 (-0.008, 0.010)	0.008 (-0.003, 0.019)	0.005 (-0.007, 0.016)	0.002 (-0.008, 0.012)
TBLH fat mass[3], kg	3.92 (3.69, 4.15)	-0.08 (-0.37, 0.20)	0.12 (-0.22, 0.47)	0.11 (-0.24, 0.47)	-0.11 (-0.43, 0.21)
TBLH fat tissue mass[3], %	31.7 (30.7, 32.7)	-0.51 (-1.79, 0.77)	-0.13 (-1.58, 1.31)	0.39 (-1.05, 1.83)	-0.86 (-2.26, 0.54)
TBLH lean mass[3], kg	8.29 (8.05, 8.53)	0.04 (-0.25, 0.34)	0.20 (-0.12, 0.53)	-0.04 (-0.36, 0.28)	0.07 (-0.22, 0.35)
WB BMC[4], g	473.1 (459.4, 486.7)	0.93 (-15.2, 17.1)	7.8 (-11.7, 27.4)	4.6 (-15.6, 24.9)	6.0 (-10.6, 22.6)
WB aBMD[4], g/cm^2	0.577 (0.568, 0.587)	0.003 (-0.008, 0.013)	0.008 (-0.004, 0.020)	0.004 (-0.010, 0.017)	0.005 (-0.006, 0.017)
WB fat mass[4], kg	4.18 (3.96, 4.40)	-0.05 (-0.33, 0.23)	0.14 (-0.19, 0.48)	0.13 (-0.24, 0.49)	-0.08 (-0.38, 0.23)
WB fat tissue mass[4], %	30.0 (29.1, 30.9)	-0.25 (-1.45, 0.94)	0.04 (-1.25, 1.33)	0.35 (-0.97, 1.67)	-0.54 (-1.76, 0.67)
WB lean mass[4], kg	9.62 (9.37, 9.88)	0.03 (-0.30, 0.35)	0.22 (-0.13, 0.57)	-0.02 (-0.37, 0.33)	0.05 (-0.25, 0.36)
Head BMC[5], g	200.6 (195.8, 205.4)	-0.95 (-7.1, 5.2)	1.0 (-4.9, 7.0)	-0.6 (-7.5, 6.4)	2.5 (-3.5, 8.5)

	Vitamin D (prenatal; postpartum IU/week)				
	**0;0**	**4200;0**	**16800;0**	**28000;0**	**28000;28000**
	**Mean (95% CI)**	**Mean Difference (95% CI)[2]**			
Head aBMD[5], g/cm^2	1.039 (1.020, 1.059)	0.004 (-0.020, 0.028)	0.009 (-0.015, 0.033)	0.009 (-0.019, 0.037)	0.017 (-0.008, 0.043)

[1]No or negligible motion artifact defined as whole body positioned within identified measurement parameters and no presence of additional artifacts within the measurement area (e.g. hand of caregiver/DXA technician). Effect estimates for between-group differences calculated from unadjusted linear regression models, with placebo as the reference group. Estimates of the 95% CIs were obtained using a bootstrap procedure with 1000 replications. aBMD; areal bone mineral density; BMC, bone mineral content; TBLH, total-body-less-head; WB, whole-body.

[2]Values represent mean difference for each vitamin D supplementation group compared to placebo.

[3]N 0;0 = 99; 4200;0 = 122; N 16800;0 = 116; 28000;0 = 109; N 28000;28000 = 110.

[4]N 0;0 = 87; 4200;0 = 108; N 16800;0 = 98; 28000;0 = 97; N 28000;28000 = 93.

[5]N 0;0 = 96; 4200;0 = 111; N 16800;0 = 102; 28000;0 = 104; N 28000;28000 = 99.

Supplementary Table 7: Effect of maternal vitamin D supplementation on offspring bone mineral content, bone mineral density and grip strength at age four years relative to placebo, by sex[1].

	Vitamin D (prenatal; postpartum IU/week)								
	**0;0**	**4200;0**		**16800;0**		**28000;0**		**28000;28000**	
	**Mean (95% CI)**	**Mean Difference (95% CI)[3]**	***P*[2]**	**Mean Difference (95% CI)[3]**	***P*[2]**	**Mean Difference (95% CI)[3]**	***P*[2]**	**Mean Difference (95% CI)[3]**	***P*[2]**
TBLH BMC (g)[4]									
Boys	285.9 (272.4, 299.4)	-9.9 (-27.3, 7.4)	0.32	-0.3 (-20.5, 19.9)	0.70	-3.9 (-23.7, 15.9)	0.64	-15.6 (-32.6, 1.3)	0.005
Girls	268.3 (256.3, 280.3)	2.2 (-14.2, 18.6)		5.0 (-12.5, 22.5)		2.7 (-16.2, 21.6)		17.2* (0.8, 33.7)	
TBLH aBMD (g/cm²)[4]									
Boys	0.446 (0.435, 0.458)	-0.008 (-0.022, 0.007)	0.34	0.002 (-0.014, 0.019)	0.69	-0.003 (-0.019, 0.014)	0.58	-0.013 (-0.028, 0.001)	0.009
Girls	0.432 (0.422, 0.441)	0.001 (-0.011, 0.014)		0.007 (-0.007, 0.021)		0.004 (-0.011, 0.019)		0.012 (-0.001, 0.026)	
TBLH fat mass (kg)[4]									
Boys	3.86 (3.52, 4.21)	-0.10 (-0.53, 0.34)	0.89	-0.03 (-0.50, 0.45)	0.49	-0.04 (-0.54, 0.45)	0.64	-0.27 (-0.70, 0.17)	0.24
Girls	4.05 (3.78, 4.33)	-0.14 (-0.51, 0.24)		0.21 (-0.26, 0.67)		0.12 (-0.37, 0.61)		0.12 (-0.34, 0.57)	
TBLH fat tissue mass (%)[4]									
Boys	29.8 (28.3, 31.3)	0.003 (-1.81, 1.82)	0.60	-0.19 (-2.08, 1.70)	0.71	0.12 (-1.99, 2.14)	0.90	-0.65 (-2.39, 1.09)	0.81
Girls	33.3 (32.2, 34.5)	-0.59 (-2.18, 0.99)		0.32 (-1.54, 2.18)		0.29 (-1.50, 2.08)		-0.35 (-2.12, 1.43)	
TBLH lean mass (kg)[4]									
Boys	8.88 (8.57, 9.19)	-0.18 (-0.59, 0.23)	0.61	-0.07 (-0.52, 0.38)	0.40	-0.22 (-0.63, 0.19)	0.53	-0.31 (-0.70, 0.08)	0.03
Girls	7.97 (7.68, 8.25)	-0.04 (-0.40, 0.32)		0.19 (-0.17, 0.55)		-0.03 (-0.43, 0.36)		0.27 (-0.09, 0.63)	
WB BMC (g)[5]									
Boys	491.4 (473.2, 509.7)	-6.6 (-26.6, 13.4)	0.67	4.2 (-21.9, 30.2)	0.99	-1.4 (-27.8, 25.0)	0.71	-9.1 (-31.4, 13.3)	0.04
Girls	462.3 (445.7, 478.9)	0.1 (-22.0, 22.2)		4.3 (-19.6, 28.2)		5.7 (-20.5, 31.8)		24.4* (3.9, 44.9)	

	Vitamin D (prenatal; postpartum IU/week)								
	0;0	4200;0		16800;0		28000;0		28000;28000	
	**Mean (95% CI)**	**Mean Difference (95% CI)[3]**	***P*[2]**	**Mean Difference (95% CI)[3]**	***P*[2]**	**Mean Difference (95% CI)[3]**	***P*[2]**	**Mean Difference (95% CI)[3]**	***P*[2]**
WB aBMD (g/cm^2)[5]									
Boys	0.589 (0.577, 0.601)	-0.002 (-0.016, 0.011)	0.85	0.008 (-0.008, 0.024)	0.83	0.001 (-0.016, 0.018)	0.70	-0.003 (-0.018, 0.011)	0.15
Girls	0.571 (0.560, 0.583)	-0.0003 (-0.016, 0.015)		0.006 (-0.010, 0.021)		0.005 (-0.011, 0.022)		0.013 (-0.002, 0.028)	
WB fat mass (kg)[5]									
Boys	4.08 (3.75, 4.41)	-0.03 (-0.43, 0.37)	0.82	0.06 (-0.42, 0.54)	0.67	0.06 (-0.40, 0.53)	0.81	-0.12 (-0.55, 0.32)	0.44
Girls	4.30 (4.04, 4.57)	-0.09 (-0.45, 0.26)		0.21 (-0.25, 0.68)		0.15 (-0.35, 0.65)		0.14 (-0.30, 0.58)	
WB fat tissue mass (%)[5]									
Boys	28.3 (27.0, 29.5)	0.09 (-1.50, 1.68)	0.67	0.12 (-1.67, 1.92)	0.90	0.49 (-1.28, 2.25)	0.92	-0.25 (-1.83, 1.33)	0.95
Girls	31.4 (30.5, 32.4)	-0.35 (-1.67, 0.97)		0.28 (-1.36, 1.92)		0.37 (-1.18, 1.92)		-0.18 (-1.72, 1.36)	
WB lean mass (kg)[5]									
Boys	10.19 (9.86, 10.52)	-0.09 (-0.49, 0.31)	0.80	-0.03 (-0.48, 0.43)	0.43	-0.16 (-0.60, 0.28)	0.63	-0.17 (-0.59, 0.25)	0.11
Girls	9.25 (8.96, 9.54)	-0.02 (-0.40, 0.36)		0.23 (-0.14, 0.61)		-0.006 (-0.44, 0.43)		0.31 (-0.05, 0.66)	
Head BMC (g)[6]									
Boys	208.8 (202.5, 215.1)	-1.7 (-9.0, 5.7)	0.87	2.1 (-5.6, 9.8)	0.50	-3.2 (-12.6, 6.2)	0.58	0.3 (-7.4, 8.0)	0.32
Girls	193.9 (187.7, 200.1)	-2.6 (-9.8, 4.6)		-1.7 (-9.0, 5.6)		0.4 (-7.5, 8.3)		5.6 (-1.8, 13.0)	
Head aBMD (g/cm^2)[6]									
Boys	1.048 (1.024, 1.072)	0.008 (-0.021, 0.037)	0.65	0.027 (-0.005, 0.058)	0.27	0.013 (-0.024, 0.050)	0.99	0.018 (-0.014, 0.049)	0.67
Girls	1.026 (1.000, 1.052)	-0.002 (-0.034, 0.030)		0.001 (-0.031, 0.033)		0.012 (-0.021, 0.046)		0.027 (-0.005, 0.059)	
Grip strength (kg)[7]									
Boys	4.64 (4.28, 5.00)	0.045 (-0.44, 0.53)	0.72	0.09 (-0.43, 0.60)	0.91	0.18 (-0.36, 0.71)	0.33	-0.08 (-0.55, 0.40)	0.53
Girls	4.35 (4.06, 4.65)	-0.07 (-0.48, 0.34)		0.13 (-0.35, 0.60)		-0.17 (-0.57, 0.24)		0.13 (-0.31, 0.58)	

[1]Effect estimates for between-group differences calculated from unadjusted linear regression models, with placebo as the reference group. Within-sex differences by intervention group were explored as sub-group analyses using individual models for each sex. Estimates of the 95% CIs were obtained using a bootstrap procedure with 1000 replications. aBMD; areal bone mineral density; BMC, bone mineral content; TBLH, total-body-less-head; WB, whole-body.

[2]Represents *P*-value for interaction terms between sex and intervention group on each outcome, calculated from unadjusted linear regression models with boys as the reference group. $P<0.05$ indicates a statistically significant difference in effect estimates for boys of that treatment group compared to placebo versus girls of that intervention group compared to placebo.

[3]Values represent mean difference for each vitamin D intervention group compared to placebo.

[4]Boys: N 0;0 = 51; N 4200;0 = 64; N 16800;0 = 63; N 28000;0 = 56; N 28000;28000 = 62. Girls: N 0;0 = 63; N 4200;0 = 62; N 16800;0 = 57; N 28000;0 = 65; N 28000;28000 = 56.

[5]Boys: N 0;0 = 46; N 4200;0 = 60; N 16800;0 = 59; N 28000;0 = 54; N 28000;28000 = 54. Girls: N 0;0 = 63; N 4200;0 = 60; N 16800;0 = 54; N 28000;0 = 60; N 28000;28000 = 55.

[6]Boys: N 0;0 = 46; N 4200;0 = 60; N 16800;0 = 62; N 28000;0 = 54; N 28000;28000 = 54. Girls: N 0;0 = 64; N 4200;0 = 60; N 16800;0 = 54; N 28000;0 = 61; N 28000;28000 = 57.

[7]Boys: N 0;0 = 53; N 4200;0 = 67; N 16800;0 = 70; N 28000;0 = 58; N 28000;28000 = 63. Girls: N 0;0 = 67; N 4200;0 = 67; N 16800;0 = 59; N 28000;0 = 67; N 28000;28000 = 59.

*Indicates significant difference relative to placebo at $P<0.05$ in sex-specific sub-group analyses.

Supplementary Table 8: Length- and height-for-age z-scores of girls from birth to 4 years of age, limited to placebo and high-dose prenatal vitamin D supplementation trial arms and stratified by study cohort.

		Vitamin D (prenatal; postpartum IU/week)		
		0;0	28000;0	28000;28000
	N	**Mean (95% CI)**		
**LAZ at birth[1]**				
Primary MDIG trial cohort[2]	249	-0.94 (-1.18, -0.71)	-1.00 (-1.25, -0.75)	-0.91 (-1.12, -0.70)
MDIG only cohort[3,4]	110	-0.86 (-1.17, -0.55)	-1.05 (-1.37, -0.73)	-0.90 (-1.19, -0.61)
BONUSKids cohort[5,6]	139	-1.01 (-1.36, -0.66)	-0.96 (-1.33, -0.60)	-0.92 (-1.23, -0.60)
**LAZ at 24 months[7]**				
Primary MDIG trial cohort[8]	323	-1.21 (-1.41, -1.02)	-1.31 (-1.53, -1.10)	-1.22 (-1.41, -1.02)
MDIG only cohort[3,9]	130	-1.27 (-1.54, -0.99)	-1.20 (-1.57, -0.84)	-1.39 (-1.71, -1.07)
BONUSKids cohort[5,10]	193	-1.17 (-1.45, -0.89)	-1.37 (-1.64, -1.10)	-1.08 (-1.32, -0.84)
**HAZ at 48 months**				
BONUSKids cohort[11]	194	-1.30 (-1.56, -1.03)	-1.33 (-1.59, -1.06)	-0.95 (-1.17, -0.73)

[1]Data is limited to anthropometric measures taken within (±) 2 days of age. BONUSKids, BONe and mUScle health in Kids study; LAZ, length-for-age z-score; HAZ, height-for-age z-score; MDIG, Maternal Vitamin D for Infant Growth Trial.

[2]N 0;0 = 82; 28000;0 = 80; N 28000;28000 = 87.

[3]Refers to MDIG trial participants who do not participate in further follow-up at 4 years of age (i.e., not included in the BONUSKids study).

[4]N 0;0 = 36; 28000;0 = 32; N 28000;28000 = 42.

[5]Refers only to MDIG trial participants who participated in further follow-up at 4 years of age (i.e., included in the BONUSKids study).

[6]N 0;0 = 46; 28000;0 = 48; N 28000;28000 = 45.

[7]Data is limited to anthropometric measures taken within (±) 30 days of age.

[8]N 0;0 = 115; 28000;0 = 102; N 28000;28000 = 106.

[9]N 0;0 = 48; 28000;0 = 35; N 28000;28000 = 47.

[10]N 0;0 = 67; 28000;0 = 67; N 28000;28000 = 59.

[11]N 0;0 = 67; 28000;0 = 67; N 28000;28000 = 60.

Supplementary Table 9: Effect of maternal vitamin D supplementation on offspring bone mineral content, bone mineral density and grip strength at age four years relative to placebo among offspring of women who were vitamin D deficient at randomization[1].

	**Vitamin D (prenatal; postpartum IU/week)**				
	**0;0**	**4200;0**	**16800;0**	**28000;0**	**28000;28000**
	**Mean (95% CI)**	**Mean Difference (95% CI)[2]**			
TBLH BMC (g)[3]	274.4 (263.6, 285.3)	-3.4 (-18.6, 11.7)	-1.6 (-19.8, 16.5)	-0.5 (-18.2, 17.1)	2.8 (-12.7, 18.4)
TBLH aBMD (g/cm^2)[3]	0.438 (0.429, 0.447)	-0.004 (-0.016, 0.008)	0.003 (-0.012, 0.017)	-0.003 (-0.017, 0.012)	0.002 (-0.011, 0.014)
TBLH fat mass (kg)[3]	3.97 (3.69, 4.25)	-0.07 (-0.45, 0.31)	-0.13 (-0.56, 0.30)	0.05 (-0.40, 0.50)	-0.08 (-0.49, 0.33)
TBLH fat tissue mass (%)[3]	31.8 (30.6, 33.0)	-0.18 (-1.75, 1.39)	-1.16 (-2.92, 0.61)	-0.03 (-1.79, 1.72)	-0.90 (-2.52, 0.72)
TBLH lean mass (kg)[3]	8.32 (8.05, 8.59)	-0.04 (-0.43, 0.35)	0.12 (-0.31, 0.53)	-0.02 (-0.40, 0.36)	0.16 (-0.21, 0.53)
WB BMC (g)[4]	473.8 (459.1, 488.5)	-0.34 (-19.4, 18.7)	-0.36 (-24.3, 23.6)	-0.24 (-23.5, 23.1)	9.8 (-10.8, 30.4)
WB aBMD (g/cm^2)[4]	0.580 (0.569, 0.590)	-0.0007 (-0.013, 0.012)	0.005 (-0.010, 0.021)	-0.001 (-0.016, 0.014)	0.004 (-0.010, 0.019)
WB fat mass (kg)[4]	4.19 (3.93, 4.46)	0.01 (-0.34, 0.36)	-0.05 (-0.46, 0.35)	0.10 (-0.34, 0.55)	0.06 (-0.33, 0.44)
WB fat tissue mass (%)[4]	30.0 (29.0, 31.1)	0.05 (-1.33, 1.42)	-0.77 (-2.25, 0.72)	0.13 (-1.45, 1.71)	-0.36 (-1.79, 1.06)
WB lean mass (kg)[4]	9.61 (9.34, 9.89)	0.04 (-0.33, 0.42)	0.15 (-0.28, 0.57)	0.01 (-0.38, 0.41)	0.26 (-0.12, 0.64)

	Vitamin D (prenatal; postpartum IU/week)				
	**0;0**	**4200;0**	**16800;0**	**28000;0**	**28000;28000**
	**Mean (95% CI)**	**Mean Difference (95% CI)[2]**			
Head BMC (g)[5]	201.5 (195.7, 207.2)	-0.7 (-7.8, 6.4)	-1.3 (-8.8, 6.2)	-3.0 (-11.1, 5.2)	1.2 (-5.9, 8.4)
Head aBMD (g/cm^2)[5]	1.039 (1.017, 1.061)	0.003 (-0.024, 0.030)	0.006 (-0.023, 0.034)	0.003 (-0.029, 0.035)	0.010 (-0.019, 0.038)
Grip strength (kg)[6]	4.45 (4.15, 4.75)	-0.06 (-0.46, 0.33)	-0.04 (-0.50, 0.42)	0.24 (-0.21, 0.69)	0.03 (-0.35, 0.41)

[1]Vitamin D deficiency defined as a 25-hydroxyvitamin D concentration <30 nmol/L at enrolment. Effect estimates for between-group differences calculated from unadjusted linear regression models, with placebo as the reference group. Estimates of the 95% CIs were obtained using a bootstrap procedure with 1000 replications. aBMD; areal bone mineral density; BMC, bone mineral content; TBLH, total-body-less-head; WB, whole-body.

[2]Values represent mean difference for each vitamin D supplementation group compared to placebo.

[3]N 0;0 = 74; 4200;0 = 77; N 16800;0 = 72; 28000;0 = 78; N 28000;28000 = 75.

[4]N 0;0 = 71; 4200;0 = 71; N 16800;0 = 69; 28000;0 = 76; N 28000;28000 = 68.

[5]N 0;0 = 71; 4200;0 = 71; N 16800;0 = 70; 28000;0 = 76; N 28000;28000 = 70.

[6]N 0;0 = 75; 4200;0 = 82; N 16800;0 = 76; 28000;0 = 80; N 28000;28000 = 78.

Supplementary Table 10: Effect of maternal vitamin D supplementation on offspring bone mineral content, bone mineral density and grip strength at age four years relative to placebo and restricted to term-born infants only[1].

	Vitamin D (prenatal; postpartum IU/week)				
	**0;0**	**4200;0**	**16800;0**	**28000;0**	**28000;28000**
	**Mean (95% CI)**	**Mean Difference (95% CI)[2]**			
TBLH BMC (g)[3]	276.2 (266.6, 285.8)	-1.6 (-14.1, 10.9)	5.6 (-8.9, 20.0)	-2.8 (-17.6, 12.1)	0.9 (-11.4, 13.3)
TBLH aBMD (g/cm^2)[3]	0.438 (0.431, 0.446)	-0.0009 (-0.011, 0.009)	0.007 (-0.004, 0.018)	-0.0009 (-0.013, 0.011)	0.0005 (-0.009, 0.011)
TBLH fat mass (kg)[3]	3.97 (3.74, 4.20)	-0.11 (-0.39, 0.17)	0.14 (-0.21, 0.48)	0.02 (-0.35, 0.40)	-0.12 (-0.44, 0.20)
TBLH fat tissue mass (%)[3]	31.7 (30.6, 32.7)	-0.32 (-1.58, 0.93)	0.15 (-1.28, 1.59)	0.22 (-1.24, 1.69)	-0.66 (-2.03, 0.71)
TBLH lean mass (kg)[3]	8.41 (8.18, 8.65)	-0.08 (-0.39, 0.22)	0.13 (-0.20, 0.47)	-0.18 (-0.50, 0.13)	-0.03 (-0.32, 0.26)
WB BMC (g)[4]	474.9 (461.7, 488.0)	0.24 (-16.0, 16.5)	9.5 (-10.1, 29.2)	-0.53 (-21.4, 20.4)	9.0 (-7.5, 25.5)
WB aBMD (g/cm^2)[4]	0.579 (0.570, 0.588)	-0.0007 (-0.013, 0.012)	0.005 (-0.010, 0.021)	-0.001 (-0.016, 0.014)	0.004 (-0.010, 0.019)
WB fat mass (kg)[4]	4.21 (3.99, 4.43)	-0.06 (-0.33, 0.22)	0.18 (-0.16, 0.53)	0.09 (-0.27, 0.46)	-0.03 (-0.36, 0.31)
WB fat tissue mass (%)[4]	30.0 (29.1, 30.9)	-0.22 (-1.36, 0.92)	0.21 (-1.09, 1.50)	0.37 (-0.94, 1.69)	-0.38 (-1.67, 0.91)
WB lean mass (kg)[4]	9.68 (9.43, 9.92)	-0.002 (-0.32, 0.32)	0.21 (-0.14, 0.56)	-1.11 (-0.46, 0.24)	0.08 (-0.23, 0.38)
Head BMC (g)[5]	200.6 (195.7, 205.5)	-1.3 (-7.4, 4.7)	2.1 (-4.2, 8.4)	-2.4 (-9.0, 4.3)	3.8 (-2.0, 9.6)

	Vitamin D (prenatal; postpartum IU/week)				
	**0;0**	**4200;0**	**16800;0**	**28000;0**	**28000;28000**
	**Mean (95% CI)**	**Mean Difference (95% CI)[2]**			
Head aBMD (g/cm^2)[5]	1.037 (1.018, 1.057)	0.004 (-0.020, 0.027)	0.019 (-0.006, 0.045)	0.008 (-0.019, 0.036)	0.025* (0.0008, 0.048)
Grip strength (kg)[6]	4.53 (4.28, 4.78)	-0.036 (-0.37, 0.30)	0.15 (-0.23, 0.53)	-0.09 (-0.46, 0.28)	-0.04 (-0.36, 0.29)

[1]Term-birth defined as delivery ≥37 weeks of gestation. Effect estimates for between-group differences calculated from unadjusted linear regression models, with placebo as the reference group. Estimates of the 95% CIs were obtained using a bootstrap procedure with 1000 replications. aBMD; areal bone mineral density; BMC, bone mineral content; TBLH, total-body-less-head; WB, whole-body.

[2]Values represent mean difference for each vitamin D supplementation group compared to placebo.

[3]N 0;0 = 101; 4200;0 = 120; N 16800;0 = 110; 28000;0 = 109; N 28000;28000 = 109.

[4]N 0;0 = 96; 4200;0 = 114; N 16800;0 = 103; 28000;0 = 103; N 28000;28000 = 101.

[5]N 0;0 = 97; 4200;0 = 114; N 16800;0 = 106; 28000;0 = 104; N 28000;28000 = 103.

[6]N 0;0 = 106; 4200;0 = 128; N 16800;0 = 118; 28000;0 = 114; N 28000;28000 = 112.

*Indicates significant difference relative to placebo at $P<0.05$.

Supplementary Table 11: Pooled analysis showing standardized mean differences for offspring total-body-less-head bone mineral content and areal density at 3-4 years of age in response to prenatal vitamin D supplementation compared to placebo[1].

	Intervention dose (IU/d)	SMD (95% CI)	*P*	Weight (%)	I^2 (%)
**TBLH BMC**					
BONUSKids[2]	4000	0.012 (-0.211, 0.234)		30.87	
COPSAC (6)	2800	0.116 (-0.161, 0.393)		19.87	
MAVIDOS (8)	1000	0.102 (-0.074, 0.279)		49.26	
**Pooled analysis**		**0.077 (-0.047, 0.201)**	0.22		0.00
**TBLH aBMD**					
BONUSKids[2]	4000	0.024 (-0.199, 0.247)		31.75	
COPSAC (6)	2800	0.000 (-0.277, 0.277)		21.52	
MAVIDOS (8)	1000	0.194 (0.018, 0.371)		46.73	
**Pooled analysis**		**0.098 (-0.037, 0.233)**	0.15		13.78

[1]Analyses were conducted using a random effects model with inverse variance weights and restricted maximum likelihood estimation. aBMD; areal bone mineral density; BMC, bone mineral content; BONUSKids, BONe and mUScle health in Kids study; COPSAC, Copenhagen Prospective Studies on Asthma in Childhood; MAVIDOS, Maternal Vitamin D Osteoporosis Study; SMD, standardized mean difference; TBLH, total-body-less-head.

[2]Intervention provided as a weekly prenatal dose of 28000 IU.

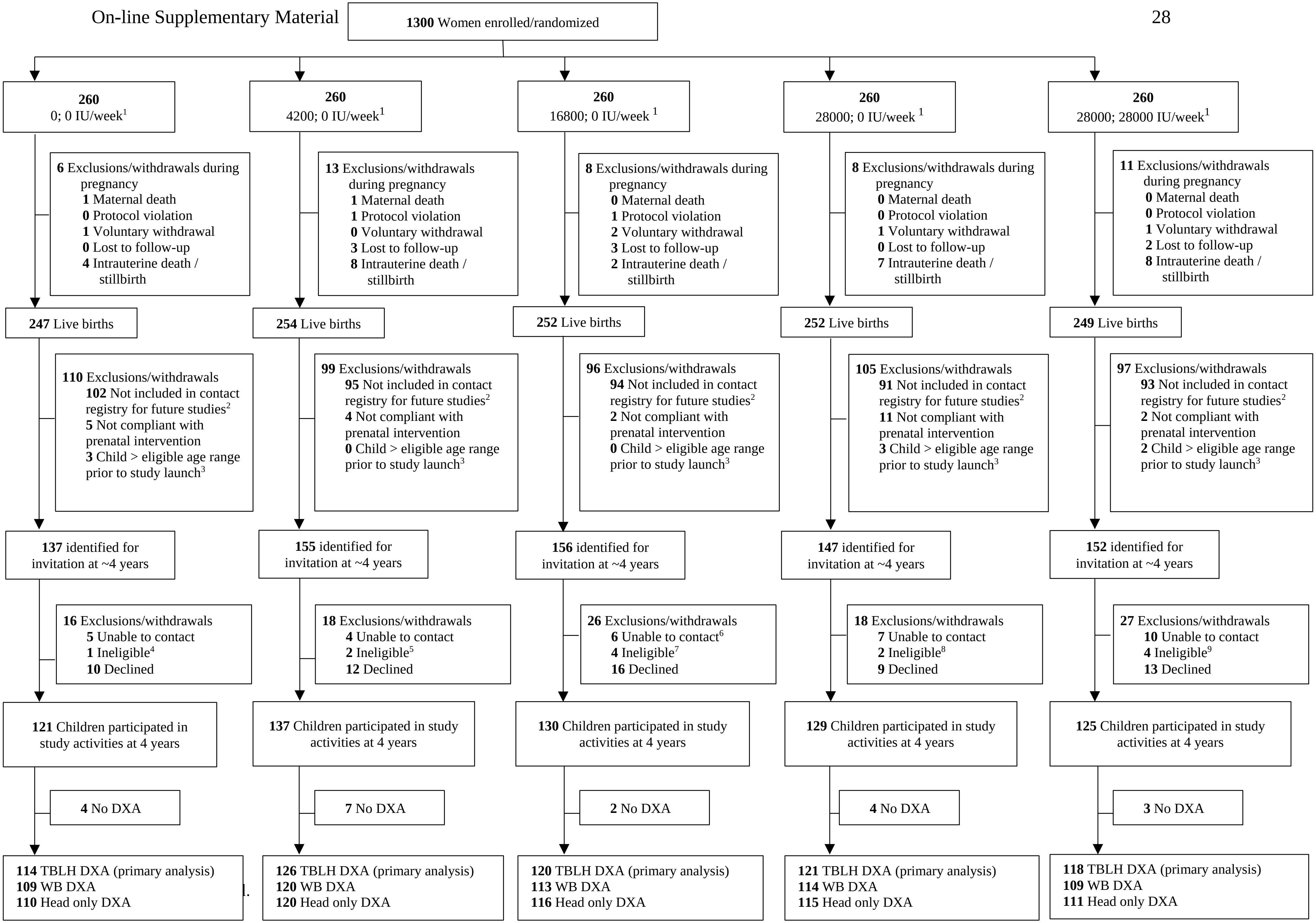

Supplementary Figure 1: CONSORT Flow Diagram of participant enrolment, random assignment and participation in study activities throughout the study by intervention group.

[1]Dose received prenatally; postpartum.

[2]Participants who either declined participation, infant died or were lost to follow-up during the postpartum period.

[3]Child was greater than 52 months of age when present study was initiated, and was therefore not contact for participation.

[4]Ineligible due to diagnosis of any developmental disorder that would render difficulty in completion of the DXA scan (*n*=1).

[5]Ineligible as could not be scheduled within the eligible age range (45-51 months of age) (*n*=2).

[6]Includes one child who was screened and eligible but unable to be contacted thereafter.

[7]Ineligible due to inability to bear weight on his/her legs (e.g. wheelchair bound) (*n*=1) or presence of current fracture or break in which his/her limb was supported by an orthopaedic cast (*n*=1), or could not be scheduled within the eligible age range (45-51 months of age) (*n*=2).

[8]Ineligible as could not be scheduled within the eligible age range (45-51 months of age) (*n*=2).

[9]Ineligible due to diagnosis of any developmental disorder that would render difficulty in completion of the DXA scan (*n*=1), unwell at time of scheduled visit (e.g. high temperature) (*n*=1) or could not be scheduled within the eligible age range (45-51 months of age) (*n*=2).

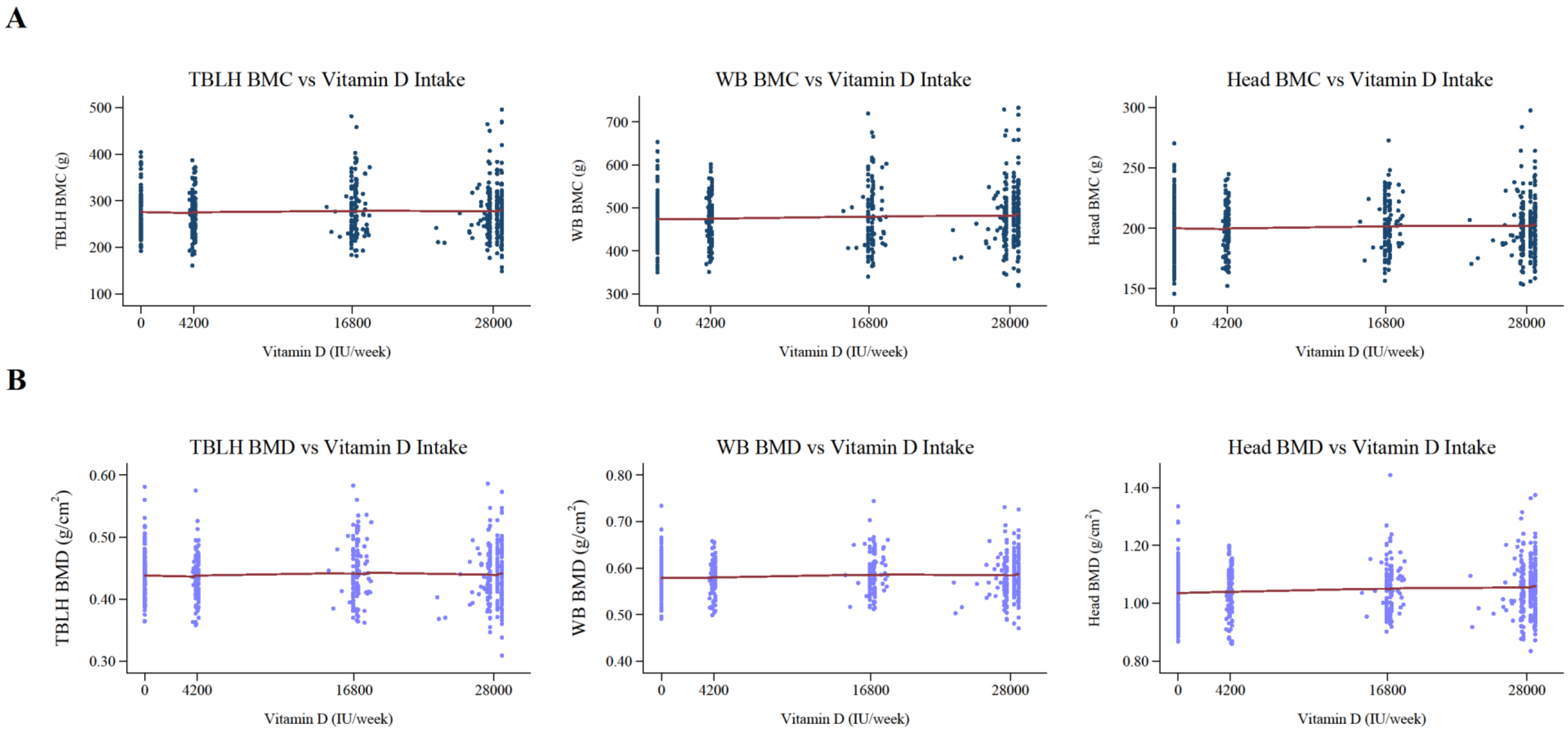

Supplementary Figure 2: Bivariate relationships between (A) BMC and prenatal supplemental vitamin D intake (IU/week) and (B) aBMD and prenatal supplemental vitamin D intake (IU/week), examined using scatter plots with locally weighted regression (LOWESS). Individual-level estimates of the vitamin D dose received during the prenatal period was estimated from manufacturer analysis of the vitamin D tablet composition and accounting for individual adherence to the intervention. Visual inspection showed a lack of a relationship between weekly prenatal supplemental vitamin D intake and each outcome. In planned sensitivity analyses, effect estimates for an association of the weekly prenatal intervention (as a continuous variable) with each outcome were consistent with the lack of a dose-response relationship. aBMD; areal bone mineral density; BMC, bone mineral content; TBLH, total-body-less-head; WB, whole-body.

Supplementary References

1. Hazell TJ, Pham TT, Jean-Philippe S, Finch SL, El Hayek J, Vanstone CA, Agellon S, Rodd CJ, Weiler HA. Vitamin D status is associated with bone mineral density and bone mineral content in preschool-aged children. J Clin Densitom. 2015;18(1):60-7.
2. Zhu K, Whitehouse AJ, Hart PH, Kusel M, Mountain J, Lye S, Pennell C, Walsh JP. Maternal vitamin D status during pregnancy and bone mass in offspring at 20 years of age: a prospective cohort study. J Bone Miner Res. 2014;29(5):1088-95.
3. Jensen SM, Molgaard C, Ejlerskov KT, Christensen LB, Michaelsen KF, Briend A. Validity of anthropometric measurements to assess body composition, including muscle mass, in 3-year-old children from the SKOT cohort. Matern Child Nutr. 2015;11(3):398-408.
4. Lorente Ramos RM, Azpeitia Arman J, Arevalo Galeano N, Munoz Hernandez A, Garcia Gomez JM, Gredilla Molinero J. Dual energy X-ray absorptimetry: fundamentals, methodology, and clinical applications. Radiologia. 2012;54(5):410-23.
5. Theodorou DJ, Theodorou SJ. Dual-energy X-ray absorptiometry in clinical practice: application and interpretation of scans beyond the numbers. Clin Imaging. 2002;26(1):43-9.
6. Brustad N, Garland J, Thorsen J, Sevelsted A, Krakauer M, Vinding RK, Stokholm J, Bønnelykke K, Bisgaard H, Chawes BL. Effect of high-dose vs standard-dose vitamin D supplementation in pregnancy on bone mineralization in offspring until age 6 Years: A prespecified secondary analysis of a double-blinded, randomized clinical trial. JAMA Pediatr. 2020;174(5):1-9.
7. Crabtree NJ, Shaw NJ, Bishop NJ, Adams JE, Mughal MZ, Arundel P, Fewtrell MS, Ahmed SF, Treadgold LA, Högler W, et al. Amalgamated reference data for size-adjusted bone densitometry measurements in 3598 children and young adults-the ALPHABET study. J Bone Miner Res. 2017;32(1):172-80.
8. Curtis EM, Cooper C, Harvey NC, On behalf of MAVIDOS Trial Investigators. Trial Report: MAVIDOS Maternal Vitamin D Osteoporosis Study. EU Clinical Trials Register. Oct 25, 2019. Available at: https://www.clinicaltrialsregister.eu/ctr-search/trial/2007-001716-23/results.
